# Supplementary material for: Reconstructing the transcriptional regulatory network of probiotic L. reuteri is enabled by transcriptomics and machine learning
Source: mSystems. 2024 Feb 13;9(3):e01257-23. doi: 10.1128/msystems.01257-23 (PMC10949432; doi:10.1128/msystems.01257-23)
Supplement: Supplemental Figures — Figures S1 to S4. [file msystems.01257-23-s0001.docx]

**Reconstructing the Transcriptional Regulatory Network of Probiotic *L. reuteri* is Enabled by Transcriptomics and Machine Learning**

Jonathan Josephs-Spaulding^1^, Akanksha Rajput^2^, Ying Hefner^2^, Richard Szubin^2^, Archana Balasubramanian^2^, Gaoyuan Li^2^, Daniel C. Zielinski^2^, Leonie Jahn^1^, Morten Sommer^1^, Patrick Phaneuf^1^, Bernhard O. Palsson^1,2*^

^1^ The Novo Nordisk Foundation Center for Biosustainability, Technical University of

Denmark, Copenhagen, Denmark

^2^ Department of Bioengineering, University of California, San Diego, La Jolla, CA, United States

^*^ Correspondence: palsson@ucsd.edu

**Supplementary Figures**


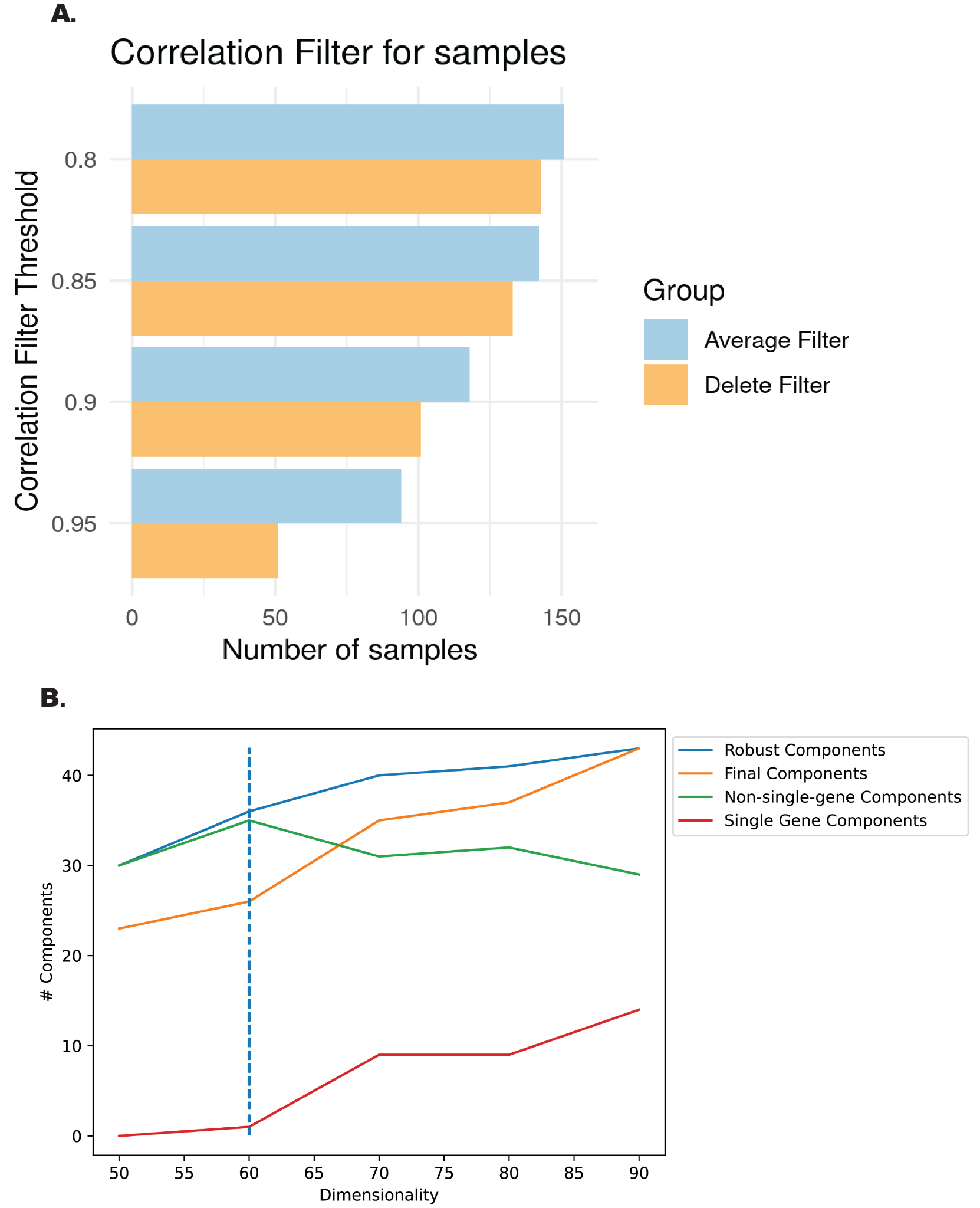


**Supplementary Figure Identification and evaluation of optimal dimensionality for independent components in the iModulon analysis of L. reuteri.** A) This figure illustrates the results of a correlation-based filtration and averaging procedure performed on gene expression data. Gene expression data were grouped according to predefined conditions, and the pairwise Pearson correlation between samples within each group was computed. For any pair of samples within a group exhibiting a correlation coefficient below a specified threshold (values used: 0.8, 0.85, 0.9, 0.95), all samples in the group were averaged to create a representative expression profile for that group. The output of this procedure is a filtered expression matrix where highly dissimilar samples have been amalgamated into single profiles. Each subfigure corresponds to a different correlation threshold used in the filtration process. B) The Python script 'get_dimension.py' was used to perform an iterative process of component extraction and evaluation to determine the dimensionality that results in the most robust and meaningful iModulon components. The figure plots the number of components identified against their respective dimensionalities. Four components are illustrated: robust components, final components, non-single-gene components, and single-gene components. Robust components represent all components found in the analysis, while final components meet a certain similarity threshold compared to the highest dimension. Non-single-gene components and single-gene components distinguish components based on whether they contain multiple genes or are driven by a single gene, respectively. A vertical dashed line indicates the optimal dimensionality that maximizes the number of final components while maintaining the most multi-gene components. This dimensionality value balances the complexity of the model (number of dimensions) and the interpretability of the components (number of multi-gene components). The results from this analysis, mainly the matrices of components (M.csv) and their activities (A.csv) at this optimal dimensionality, serve as the basis for subsequent iModulon characterization and interpretation.


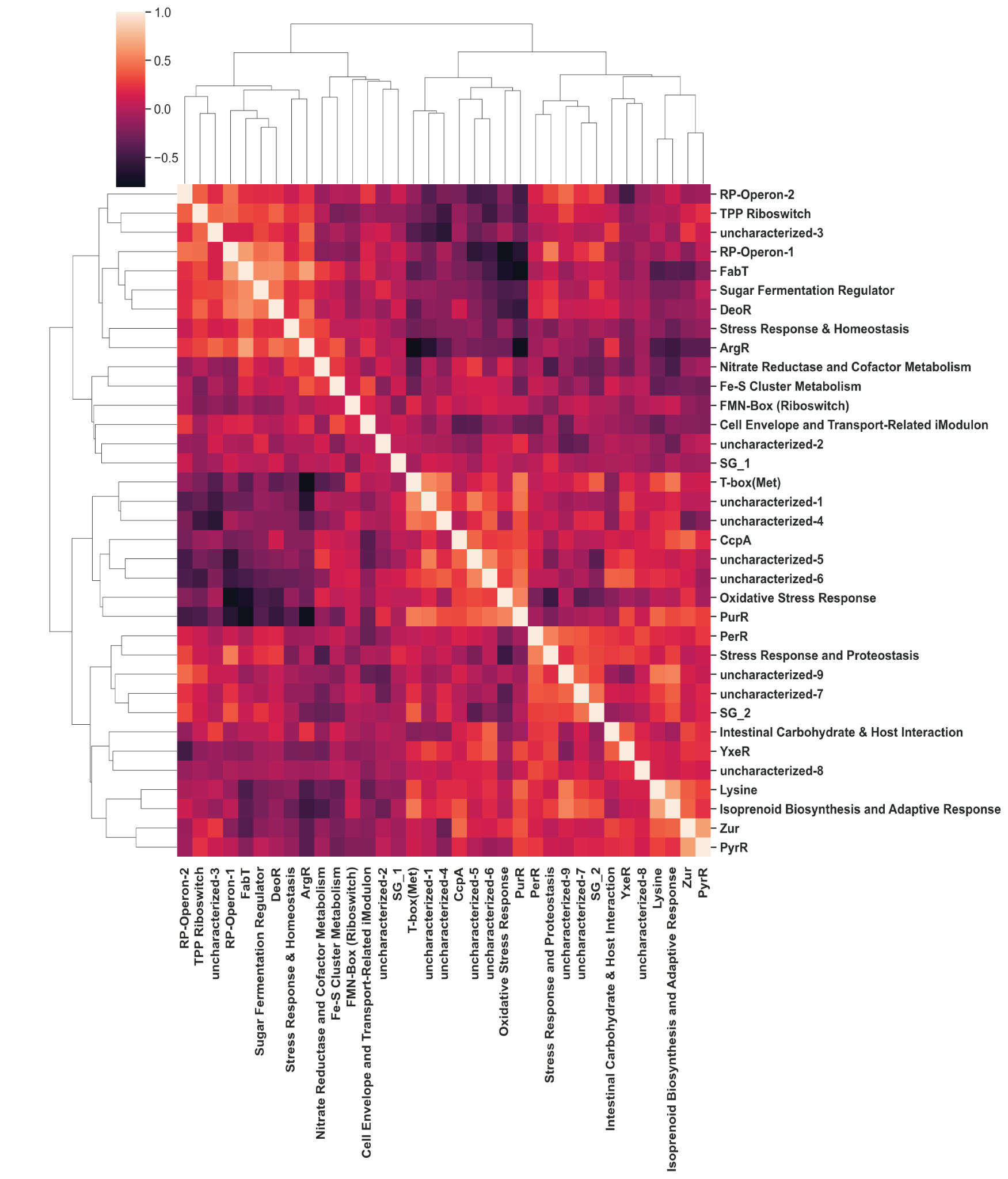


**Supplementary Figure S2: IModulon Correlation Heatmap.** The figure displays a cluster map representing the correlation among selected iModulons in L. reuteri. This heatmap utilizes hierarchical clustering to group iModulons based on similarities in their gene expression patterns across various conditions. The dendrogram and bold labels emphasize strong correlations, providing insight into potential shared or complementary roles in cellular responses.


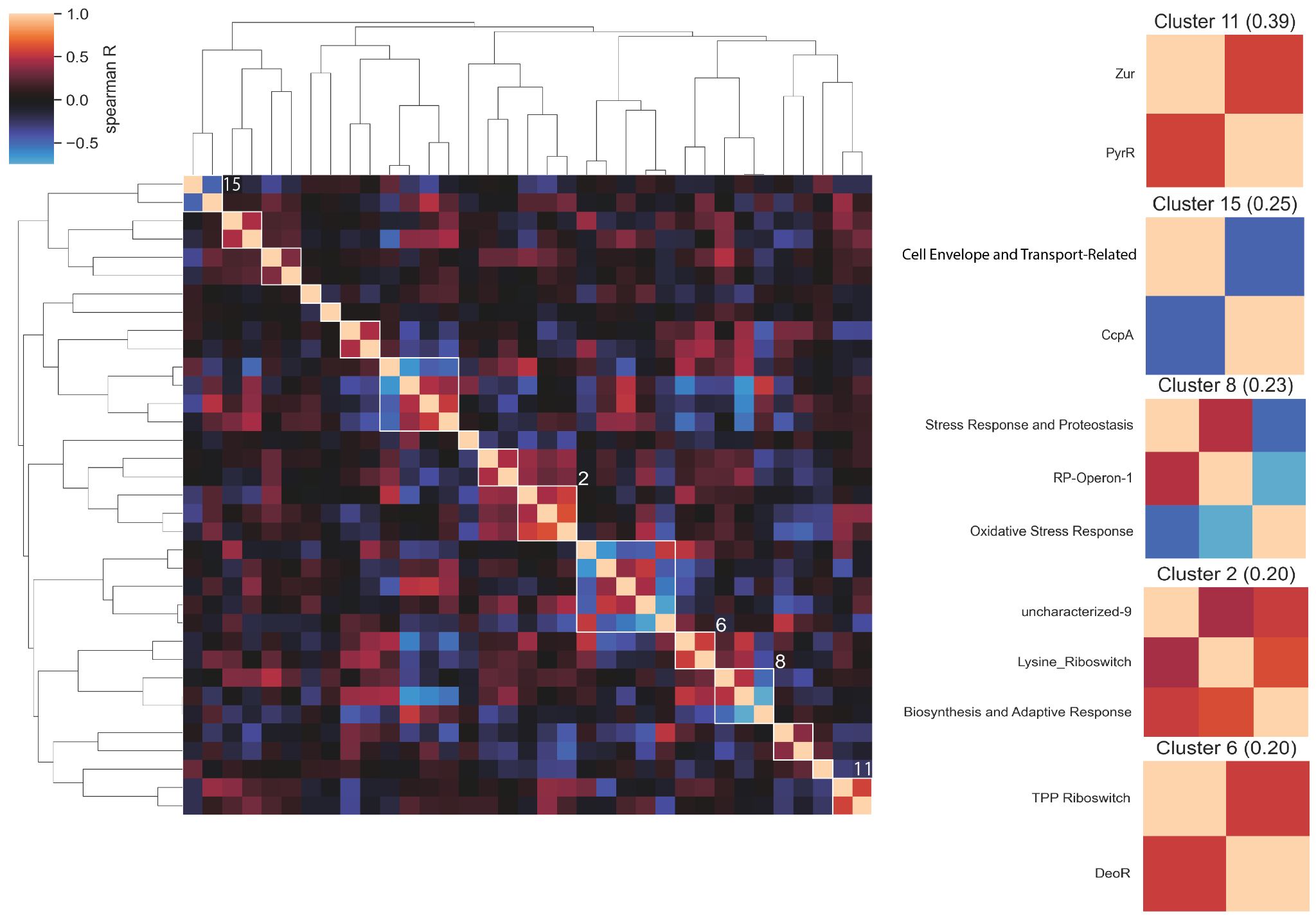


**Supplementary Figure S3: Hierarchical clustering of the five most significant iModulon clusters in L. reuteri.** Each sub-figure within the set represents a distinct iModulon cluster and its corresponding activity profile across various conditions. These visuals highlight the clusters that exhibit the most substantial and consistent patterns, providing insight into the potential co-regulation of iModulons and their collective impact on *L. reuteri's* cellular response


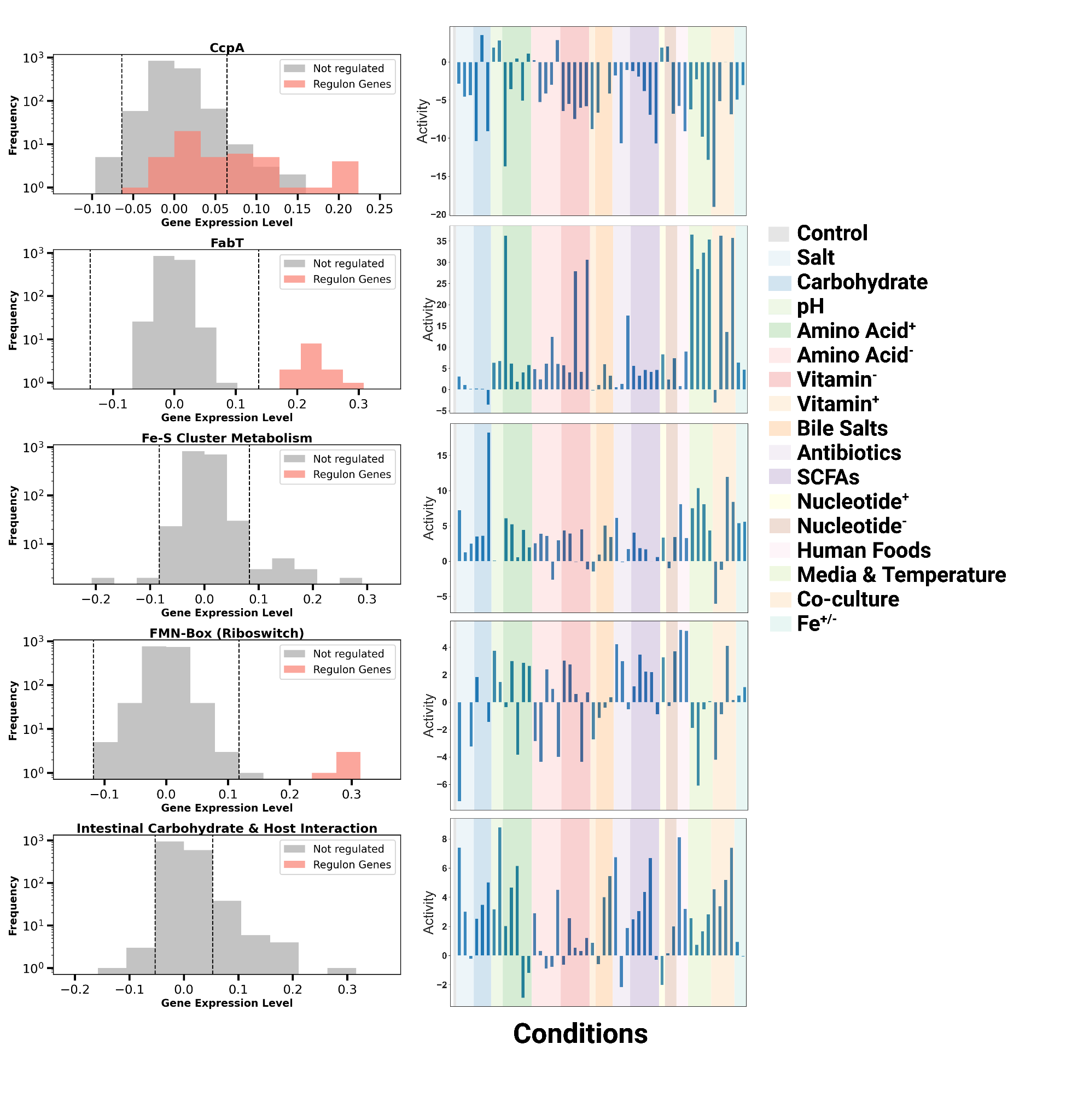


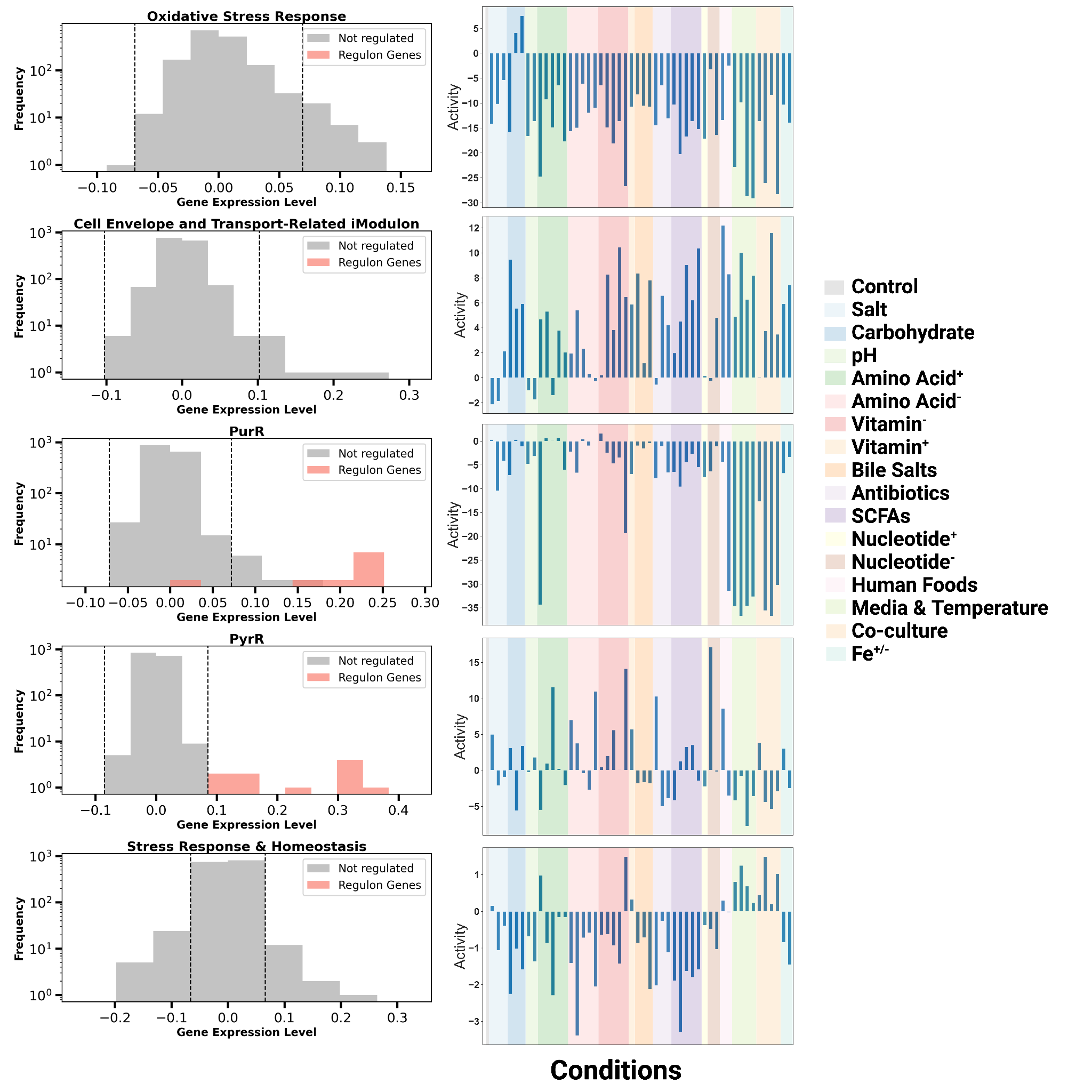


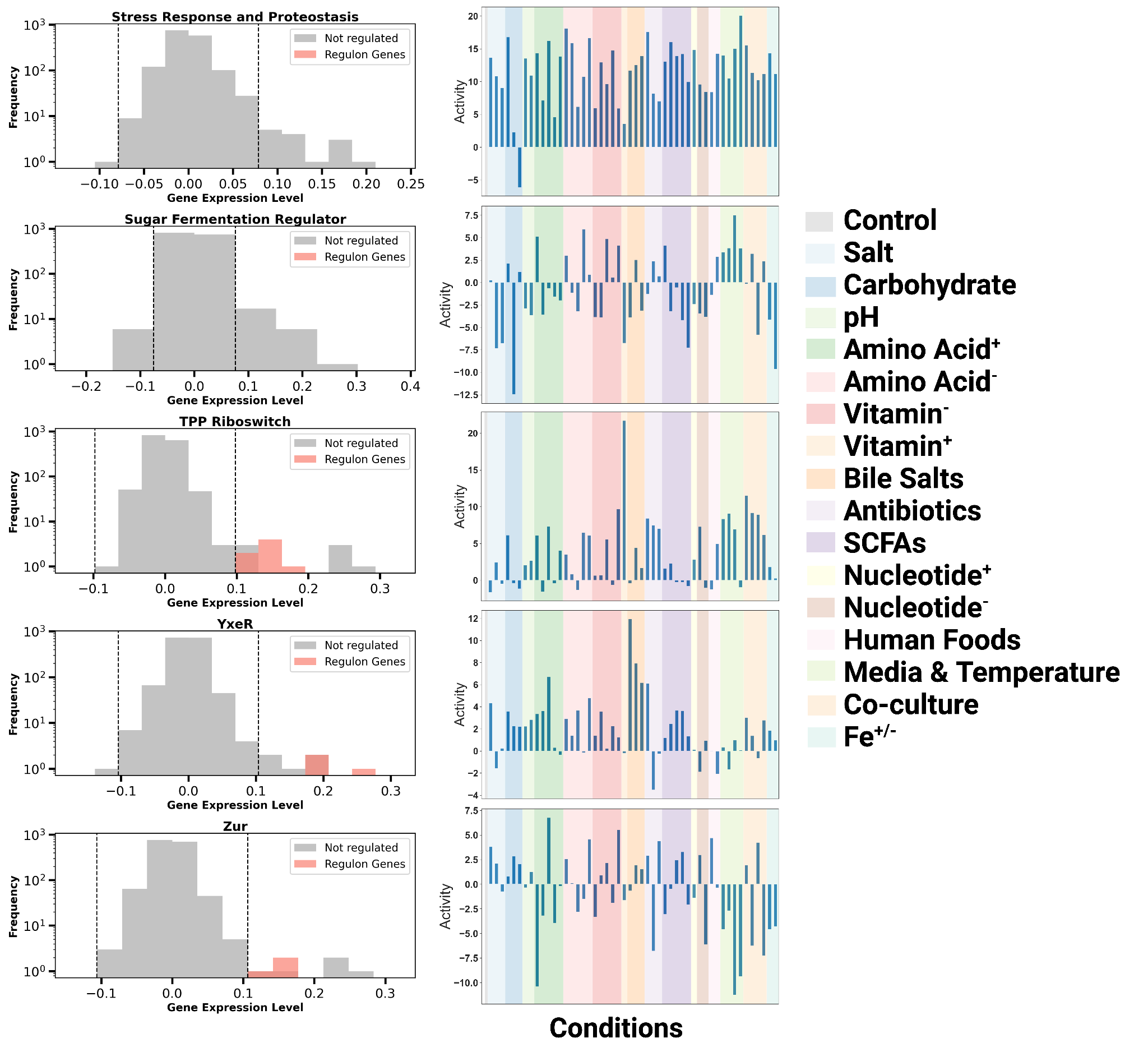


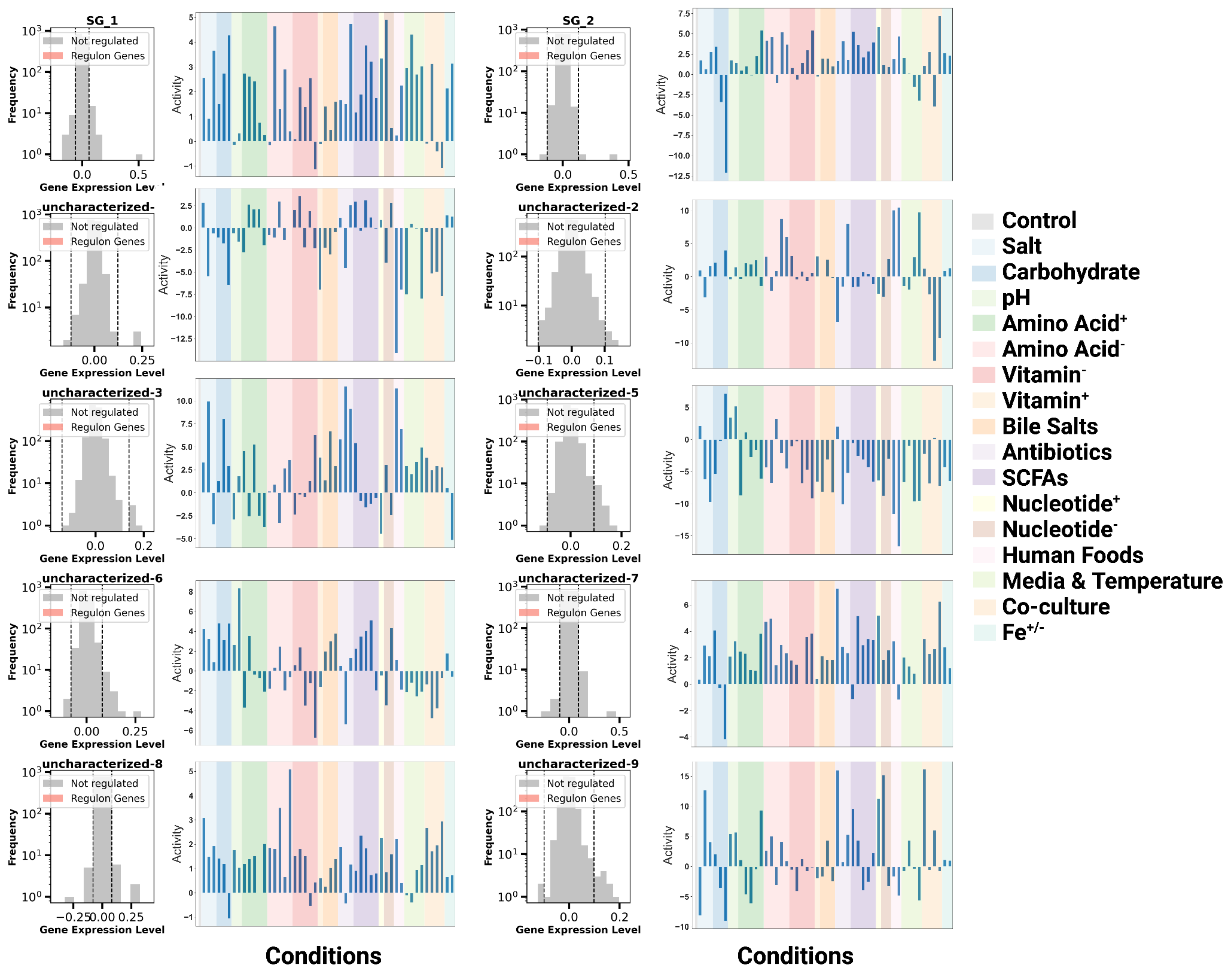


**Supplementary Figure S4**: Remaining 25 iModulons that were identified in the LatoPRECISE compendium
