# Supplementary material for: Reconstructing the transcriptional regulatory network of probiotic L. reuteri is enabled by transcriptomics and machine learning
Source: mSystems. 2024 Feb 13;9(3):e01257-23. doi: 10.1128/msystems.01257-23 (PMC10949432; doi:10.1128/msystems.01257-23)
Supplement: Supplemental Methods — Utilization of ChatGPT for iModulon characterization. [file msystems.01257-23-s0002.pdf]

Reconstructing the Transcriptional Regulatory Network of Probiotic *L. reuteri* is Enabled by Transcriptomics and Machine Learning

Jonathan Josephs-Spaulding[1], Akanksha Rajput[2], Ying Hefner[2], Richard Szubin[2], Archana Balasubramanian[2], Gaoyuan Li[2], Daniel C. Zielinski[2], Leonie Jahn[1], Morten Sommer[1], Patrick Phaneuf[1], Bernhard O. Palsson[1,2*]
[1] The Novo Nordisk Foundation Center for Biosustainability, Technical University of Denmark, Copenhagen, Denmark
[2] Department of Bioengineering, University of California, San Diego, La Jolla, CA, United States
[*] Correspondence: palsson@ucsd.edu

Supplementary Methods/Results

Utilization of ChatGPT for iModulon Characterization

In this study, ChatGPT 3.5 (August 3 Version) played an important role in characterizing iModulons within regulatory networks. Our approach utilized the natural language processing capabilities of ChatGPT to generate iModulon names and propose potential biological functions based on the provided genetic data. The process began with collecting comprehensive gene data, encompassing gene weights, genomic positions, strand information, gene names, Cluster of Orthologous Groups (COG) classifications, accessions, and regulator associations for each gene within each Uncharacterized iModulon.

ChatGPT was then introduced into the method, serving as a tool for interaction with the gene data. A prompt was formulated to guide ChatGPT in generating distinctive names for each iModulon, capitalizing on the unique characteristics of its constituent genes. Subsequently, ChatGPT undertook the task of predicting potential biological functions for each iModulon by analyzing the functional annotations associated with its constituent genes. In cases where the available data presented challenges or ambiguities, and ChatGPT could not confidently assign a name or function, the iModulon was designated as 'Uncharacterized.' This comprehensive yet adaptable approach empowered us to systematically and informatively characterize iModulons within the regulatory network, leveraging the capabilities of ChatGPT to generate biologically relevant descriptions for these essential regulatory modules. All conversations, including data input, can be found below.

Uncharacterized iModulon 2
Jonathan Josephs-Spaulding:
Given the following information, I would like to characterize 'uncharacterized iModulon 2'. Please suggest a name to describe this iModulon and a potential biological function. However, if you cannot make a decision due to limited information, please state that this iModulon is 'Uncharacterized':
,gene_weight,start,end,strand,gene_name,COG,accession,regulator
LMB90_RS00440,0.0676726510387065,1021,1365,LMB90_RS00440,Op49,Transcription,NZ_JAJGVJ010000030.1,
LMB90_RS00485,0.0802028289855046,9381,9782,-,psiE,Op277,Function Unknown,NZ_JAJGVJ010000030.1,
LMB90_RS00980,-0.147548721943623,126148,126414,LMB90_RS00980,Op1900,"Translation, Ribosomal Structure, & Biogenesis",NZ_JAJGVJ010000030.1,
LMB90_RS01260,-0.0751596265032376,12901,13755,LMB90_RS01260,Op374,Function Unknown,NZ_JAJGVJ010000039.1,
LMB90_RS01335,0.1075046375410796,28084,28356,LMB90_RS01335,Op707,No COG Annotation,NZ_JAJGVJ010000039.1,
LMB90_RS01370,-0.1028589662427037,32812,33762,LMB90_RS01370,Op813,Inorganic Ion Transport & Metabolism,NZ_JAJGVJ010000039.1,
LMB90_RS01560,0.077704519011353,30771,31346,LMB90_RS01560,Op763,Function Unknown,NZ_JAJGVJ010000044.1,
LMB90_RS01700,-0.0694030267135756,13503,14279,-,LMB90_RS01700,Op392,No COG Annotation,NZ_JAJGVJ010000045.1,
LMB90_RS01725,0.0880455005289851,20887,21225,-,LMB90_RS01725,Op561,"Intracellular Trafficking, Secretion, & Vesicular Transport",NZ_JAJGVJ010000045.1,
LMB90_RS01790,-0.1076757736304939,31800,32066,LMB90_RS01790,Op791,Signal Transduction Mechanisms,NZ_JAJGVJ010000045.1,
LMB90_RS01870,-0.1061925150427289,47577,48053,-,LMB90_RS01870,Op1085,Nucleotide Transport & Metabolism,NZ_JAJGVJ010000045.1,
LMB90_RS02655,-0.0915323735153796,331,579,-,LMB90_RS02655,Op36,Function Unknown,NZ_JAJGVJ010000048.1,
LMB90_RS02705,0.1289678637305335,10508,11194,-,LMB90_RS02705,Op307,No COG Annotation,NZ_JAJGVJ010000048.1,
LMB90_RS02930,0.138539595108138,36219,36674,LMB90_RS02930,Op882,No COG Annotation,NZ_JAJGVJ010000050.1,
LMB90_RS03860,-0.068005989642956,1733,2365,LMB90_RS03860,Op64,Function Unknown,NZ_JAJGVJ010000052.1,
LMB90_RS04100,-0.0899904709111625,5090,5575,-,LMB90_RS04100,Op156,Energy Production & Conversion,NZ_JAJGVJ010000053.1,
LMB90_RS04420,-0.0866885602504942,51796,52218,-,LMB90_RS04420,Op1154,Function Unknown,NZ_JAJGVJ010000055.1,
LMB90_RS04525,-0.0992089170174711,72987,73196,LMB90_RS04525,Op1469,No COG Annotation,NZ_JAJGVJ010000055.1,
LMB90_RS05090,-0.1098969328006575,51231,51602,-,mscL,Op1144,Cell Wall/Membrane/Envelope Biogenesis,NZ_JAJGVJ010000056.1,
LMB90_RS05430,-0.0669623944025719,35859,36905,LMB90_RS05430,Op872,Cell Motility,NZ_JAJGVJ010000058.1,
LMB90_RS05475,-0.075363095258816,42363,42758,LMB90_RS05475,Op993,No COG Annotation,NZ_JAJGVJ010000058.1,
LMB90_RS05765,0.0762696580562321,96869,97279,-,LMB90_RS05765,Op1726,"Translation, Ribosomal Structure, & Biogenesis",NZ_JAJGVJ010000058.1,
LMB90_RS05830,-0.0792480407653484,7043,7477,-,LMB90_RS05830,Op207,Cell Motility,NZ_JAJGVJ010000059.1,
LMB90_RS05840,-0.096684061082916,7785,8855,-,LMB90_RS05840,Op231,Cell Motility,NZ_JAJGVJ010000059.1,
LMB90_RS06330,0.1752119884739333,13951,14247,LMB90_RS06330,Op403,No COG Annotation,NZ_JAJGVJ010000060.1,
LMB90_RS06425,-0.0767105485470922,30914,31546,LMB90_RS06425,Op766,"Replication, Recombination, & Repair",NZ_JAJGVJ010000060.1,
LMB90_RS07085,-0.0710181928412689,36152,36559,LMB90_RS07085,Op879,Signal Transduction Mechanisms,NZ_JAJGVJ010000062.1,
LMB90_RS07335,-0.084316321645414,88550,89086,LMB90_RS07335,Op1652,No COG Annotation,NZ_JAJGVJ010000062.1,
LMB90_RS07475,-0.1300254119053978,116,520,-,LMB90_RS07475,Op26,No COG Annotation,NZ_JAJGVJ010000063.1,
LMB90_RS07495,0.0901242892833819,5819,7501,LMB90_RS07495,Op179,Cell Wall/Membrane/Envelope Biogenesis,NZ_JAJGVJ010000063.1,"CcpA,MalR3"
LMB90_RS07710,-0.0791425267454345,84953,85594,-,LMB90_RS07710,Op1616,Nucleotide Transport & Metabolism,NZ_JAJGVJ010000063.1,
LMB90_RS07735,-0.1823086800255885,4451,4765,LMB90_RS07735,Op145,Inorganic Ion Transport & Metabolism,NZ_JAJGVJ010000064.1,
LMB90_RS08170,-0.0815496837797497,9030,9605,-,LMB90_RS08170,Op268,No COG Annotation,NZ_JAJGVJ010000065.1,
LMB90_RS08245,-0.0684123321301625,22526,23566,rfbB,Op597,Coenzyme Transport & Metabolism,NZ_JAJGVJ010000065.1,
LMB90_RS08385,0.0667467956566895,58843,59265,LMB90_RS08385,Op1257,"Replication, Recombination, & Repair",NZ_JAJGVJ010000065.1,
LMB90_RS08405,-0.1454857974783329,61634,61915,LMB90_RS08405,Op1299,Carbohydrate Transport & Metabolism,NZ_JAJGVJ010000065.1,
LMB90_RS08425,-0.0911295005518642,1835,2695,LMB90_RS08425,Op73,No COG Annotation,NZ_JAJGVJ010000066.1,
LMB90_RS09060,-0.0717510257738904,131721,132476,-,LMB90_RS09060,Op1915,Amino Acid Transport & Conversion,NZ_JAJGVJ010000066.1,
LMB90_RS09165,0.0884859334878826,151999,152265,-,LMB90_RS09165,Op1948,Function Unknown,NZ_JAJGVJ010000066.1,

LMB90_RS09330,0.0885882076164644,35491,36183,LMB90_RS09330,Op869,Function Unknown,NZ_JAJGVJ010000067.1,PLP-aminotransferase
LMB90_RS09335,0.2373679101761809,36170,36493,LMB90_RS09335,Op880,Transcription,NZ_JAJGVJ010000067.1,
LMB90_RS09425,-0.1343144071975505,49891,50199,LMB90_RS09425,Op1119,Signal Transduction Mechanisms,NZ_JAJGVJ010000067.1,
LMB90_RS09910,0.1015006944039989,31270,32100,-,LMB90_RS09910,Op776,Function Unknown,NZ_JAJGVJ010000069.1,
LMB90_RS10095,-0.1639883876028717,62383,62598,-,LMB90_RS10095,Op1310,Function Unknown,NZ_JAJGVJ010000069.1,

ChatGPT:
Name: Stress Response and Homeostasis iModulon

Biological Function: The 'Stress Response and Homeostasis iModulon likely plays a central role in responding to various environmental stressors and regulating metabolic processes in the cell. This iModulon incorporates genes associated with diverse functions:

1. **Stress Response**: Genes like mscL, which are involved in mechanosensing and respond to osmotic or mechanical stress, suggest a primary role in environmental stress response mechanisms.

2. **Metabolic Regulation**: The presence of genes related to riboflavin (Vitamin B2) synthesis (rfbB) indicates a role in regulating metabolic pathways, specifically those related to cofactor synthesis.

3. **RNA Modification**: The gene psiE, involved in the catabolism of uridine and likely post-transcriptional modification of RNA, suggests a role in fine-tuning gene expression and RNA processing.

4. **Signal Transduction**: Genes related to signal transduction mechanisms indicate a role in sensing and responding to extracellular signals, potentially coordinating cellular responses.

5. **Cell Wall/Membrane/Envelope Biogenesis**: Genes associated with cell wall functions suggest a role in maintaining cell integrity and structure.

6. **Cell Motility**: The presence of genes associated with cell motility implies a potential involvement in cellular movement or response to environmental cues.

7. **Replication/Repair**: Genes associated with replication and repair suggest a role in maintaining genomic integrity, especially under stress conditions.

8. **Transcription Regulation**: PLP-aminotransferase is identified as a regulator of a gene, indicating involvement in transcriptional regulation.

This iModulon appears to function as a hub for integrating signals from the environment and regulating various cellular processes to adapt to changing conditions. Its diverse gene composition suggests a versatile role in cellular homeostasis, stress adaptation, and metabolic control. Further experimental studies would be necessary to validate its precise functions and regulatory mechanisms.

Uncharacterized iModulon 3
Jonathan Josephs-Spaulding:
Given the following information, I would like to characterize 'uncharacterized iModulon 3'. Please suggest a name to describe this iModulon and a potential biological function. However, if you cannot make a decision due to limited information, please state that this iModulon is 'Uncharacterized':
,gene_weight,start,end,strand,gene_name,COG,accession,regulator
LMB90_RS01645,0.1074632334794471,3924,4286,LMB90_RS01645,Op133,Function Unknown,NZ_JAJGVJ010000045.1,
LMB90_RS01890,0.1046033293309631,49900,50514,recU,Op1121,"Replication, Recombination, & Repair",NZ_JAJGVJ010000045.1, LMB90_RS02450,0.1234796144704556,60508,61857,LMB90_RS02450,Op1282,Carbohydrate Transport & Metabolism,NZ_JAJGVJ010000046.1,MalR
LMB90_RS04490,0.2029021811052606,66229,66840,LMB90_RS04490,Op1369,Cell Wall/Membrane/Envelope Biogenesis,NZ_JAJGVJ010000055.1, LMB90_RS05270,0.1043639034267173,891,1079,LMB90_RS05270,Op47,No COG Annotation,NZ_JAJGVJ010000058.1, LMB90_RS05310,0.2475020506556878,9917,10711,LMB90_RS05310,Op296,Cell Wall/Membrane/Envelope Biogenesis,NZ_JAJGVJ010000058.1,
LMB90_RS05320,0.210642312308865,12897,13601,LMB90_RS05320,Op373,Cell Wall/Membrane/Envelope Biogenesis,NZ_JAJGVJ010000058.1, LMB90_RS08335,0.1057566856806947,45502,45759,LMB90_RS08335,Op1053,Coenzyme

Transport & Metabolism,NZ_JAJGVJ010000065.1,
LMB90_RS08870,0.1068720819324135,94523,95551,-,LMB90_RS08870,Op1707,"Intracellular Trafficking, Secretion, & Vesicular Transport",NZ_JAJGVJ010000066.1, LMB90_RS10020,0.1506111774448739,49948,50310,-,LMB90_RS10020,Op1125,Energy Production & Conversion,NZ_JAJGVJ010000069.1,

ChatGPT:
Name: "Cell Envelope and Transport-Related iModulon"

Potential Biological Function: This iModulon appears to be involved in various cellular processes related to cell envelope biogenesis and transport. It includes genes associated with "Cell Wall/Membrane/Envelope Biogenesis," suggesting a role in maintaining cell structure and integrity. Additionally, it contains a gene involved in "Coenzyme Transport & Metabolism," indicating a potential role in the transport of coenzymes, essential for various metabolic pathways. The presence of a gene related to "Energy Production & Conversion" suggests a possible involvement in energy metabolism. However, the precise biological function of this iModulon requires further investigation to uncover its specific regulatory mechanisms and cellular pathways.

Uncharacterized iModulon 6
Jonathan Josephs-Spaulding:
Given the following information, I would like to characterize 'uncharacterized iModulon 6'. Please suggest a name to describe this iModulon and a potential biological function. However, if you cannot make a decision due to limited information, please state that this iModulon is 'Uncharacterized': ,gene_weight,start,end,strand,gene_name,COG,accession,regulator
LMB90_RS03020,0.0832927391357793,56944,57363,rpsL,Op1230,"Translation, Ribosomal Structure, & Biogenesis",NZ_JAJGVJ010000050.1, LMB90_RS03025,0.0758093150266566,57384,57854,rpsG,Op1236,"Translation, Ribosomal Structure, & Biogenesis",NZ_JAJGVJ010000050.1,
LMB90_RS03035,0.1254034016517191,60512,60820,rpsJ,Op1283,"Translation, Ribosomal Structure, & Biogenesis",NZ_JAJGVJ010000050.1, LMB90_RS03040,0.1261965714668332,60869,61528,rplC,Op1288,"Translation, Ribosomal Structure, & Biogenesis",NZ_JAJGVJ010000050.1,
LMB90_RS03045,0.1336437605268479,61550,62173,rplD,Op1297,"Translation, Ribosomal Structure, & Biogenesis",NZ_JAJGVJ010000050.1, LMB90_RS03050,0.1374413137513288,62173,62469,rplW,Op1306,"Translation, Ribosomal Structure, & Biogenesis",NZ_JAJGVJ010000050.1,
LMB90_RS03055,0.1356614734282222,62495,63340,rplB,Op1316,"Translation, Ribosomal Structure, & Biogenesis",NZ_JAJGVJ010000050.1, LMB90_RS03060,0.1292915936676724,63381,63659,rpsS,Op1325,"Translation, Ribosomal Structure, & Biogenesis",NZ_JAJGVJ010000050.1,
LMB90_RS03065,0.1287044075059738,63677,64024,rplV,Op1331,"Translation, Ribosomal Structure, & Biogenesis",NZ_JAJGVJ010000050.1, LMB90_RS03070,0.1182808996196664,64037,64702,rpsC,Op1335,"Translation, Ribosomal Structure, & Biogenesis",NZ_JAJGVJ010000050.1, LMB90_RS03075,0.1198314412369112,64706,65140,rplP,Op1343,"Translation, Ribosomal Structure, & Biogenesis",NZ_JAJGVJ010000050.1,
LMB90_RS03080,0.1214627135905744,65130,65336,rpmC,Op1351,"Translation, Ribosomal Structure, & Biogenesis",NZ_JAJGVJ010000050.1, LMB90_RS03085,0.1233438336492986,65361,65627,rpsQ,Op1354,"Translation, Ribosomal Structure, & Biogenesis",NZ_JAJGVJ010000050.1, LMB90_RS03090,0.128233840454231,65697,66065,rplN,Op1360,"Translation, Ribosomal Structure, & Biogenesis",NZ_JAJGVJ010000050.1,
LMB90_RS03095,0.133812366609405,66101,66409,rplX,Op1364,"Translation, Ribosomal Structure, & Biogenesis",NZ_JAJGVJ010000050.1, LMB90_RS03100,0.1337779485022276,66438,66980,rplE,Op1372,"Translation, Ribosomal Structure, & Biogenesis",NZ_JAJGVJ010000050.1,
LMB90_RS03105,0.1341972640058209,66996,67181,LMB90_RS03105,Op1378,"Translation, Ribosomal Structure, & Biogenesis",NZ_JAJGVJ010000050.1, LMB90_RS03110,0.1274321335087248,67214,67612,rpsH,Op1382,"Translation, Ribosomal Structure, & Biogenesis",NZ_JAJGVJ010000050.1, LMB90_RS03115,0.1318134704183286,67642,68178,rplF,Op1390,"Translation, Ribosomal Structure, & Biogenesis",NZ_JAJGVJ010000050.1,
LMB90_RS03120,0.12919165779751,68241,68597,rplR,Op1400,"Translation, Ribosomal Structure, & Biogenesis",NZ_JAJGVJ010000050.1, LMB90_RS03125,0.1250432771074615,68619,69128,rpsE,Op1404,"Translation, Ribosomal Structure, & Biogenesis",NZ_JAJGVJ010000050.1,
LMB90_RS03130,0.1226074082241952,69144,69326,rpmD,Op1409,"Translation, Ribosomal Structure, & Biogenesis",NZ_JAJGVJ010000050.1, LMB90_RS03135,0.1251331008651087,69358,69792,rplO,Op1413,"Translation, Ribosomal Structure, & Biogenesis",NZ_JAJGVJ010000050.1,
LMB90_RS03140,0.1260711047241463,69794,71110,secY,Op1421,"Intracellular Trafficking, Secretion, & Vesicular Transport",NZ_JAJGVJ010000050.1, LMB90_RS03145,0.1254253321866579,71110,71769,LMB90_RS03145,Op1438,Nucleotide Transport & Metabolism,NZ_JAJGVJ010000050.1, LMB90_RS03150,0.0907317435453244,71903,72121,infA,Op1452,"Translation, Ribosomal Structure, & Biogenesis",NZ_JAJGVJ010000050.1,
LMB90_RS03155,0.0965725594650109,72158,72277,rpmJ,Op1455,"Translation, Ribosomal Structure, & Biogenesis",NZ_JAJGVJ010000050.1, LMB90_RS03160,0.0931292787834273,72309,72674,rpsM,Op1459,"Translation, Ribosomal Structure, & Biogenesis",NZ_JAJGVJ010000050.1,

LMB90_RS03165,0.0823871928553436,72709,73098,rpsK,Op1466,"Translation, Ribosomal Structure, & Biogenesis",NZ_JAJGVJ010000050.1,
LMB90_RS03170,0.0886734709897455,73184,74128,LMB90_RS03170,Op1472,Transcription,NZ_JAJGVJ010000050.1,
LMB90_RS03175,0.0753337579291158,74163,74546,rplQ,Op1485,"Translation, Ribosomal Structure, & Biogenesis",NZ_JAJGVJ010000050.1, LMB90_RS05070,0.1653607903873125,49340,49840,rplJ,Op1114,"Translation, Ribosomal Structure, & Biogenesis",NZ_JAJGVJ010000056.1,L10_leader
LMB90_RS05075,0.1493060949281395,49903,50268,rplL,Op1122,"Translation, Ribosomal Structure, & Biogenesis",NZ_JAJGVJ010000056.1,L10_leader LMB90_RS05680,0.0756591352635798,83545,84057,infC,Op1601,"Translation, Ribosomal Structure, & Biogenesis",NZ_JAJGVJ010000058.1,L20_leader
LMB90_RS06450,0.0779731271527819,35984,36253,rpsO,Op876,"Translation, Ribosomal Structure, & Biogenesis",NZ_JAJGVJ010000060.1,S15

ChatGPT:

Name: "Ribosomal Protein iModulon"

Potential Biological Function: This iModulon is primarily associated with genes related to "Translation, Ribosomal Structure, & Biogenesis." It contains genes encoding various ribosomal proteins, such as rpsL, rpsG, rplC, rplD, rplW, rplB, rpsS, rplV, rpsC, rplP, rpmC, rpsQ, rplN, rplX, rplE, rpsH, rplF, rplR, rpsE, rpmD, rplO, secY, infA, rpmJ, rpsM, rpsK, rplQ, rplJ, and rplL.

Given the prevalence of ribosomal proteins in this iModulon, it likely plays a crucial role in ribosome assembly, a fundamental process in protein synthesis. Ribosomal proteins are essential for the structure and function of ribosomes, which are responsible for protein translation in the cell. Therefore, this iModulon is likely involved in regulating the production and assembly of ribosomes, which is critical for cellular growth and protein synthesis.

Uncharacterized iModulon 9

Jonathan Josephs-Spaulding:

Given the following information, I would like to characterize 'uncharacterized iModulon 9'. Please suggest a name to describe this iModulon and a potential biological function. However, if you cannot make a decision due to limited information, please state that this iModulon is 'Uncharacterized': ,gene_weight,start,end,strand,gene_name,COG,accession,regulator
LMB90_RS01270,0.1391487286079943,14528,15418,LMB90_RS01270,Op418,Amino Acid Transport & Conversion,NZ_JAJGVJ010000039.1, LMB90_RS01275,0.2831342484545917,15487,16176,-,narI,Op440,Energy Production & Conversion,NZ_JAJGVJ010000039.1, LMB90_RS01280,0.3017630869232899,16169,16747,-,narJ,Op458,Energy Production & Conversion,NZ_JAJGVJ010000039.1, LMB90_RS01285,0.3026123141398768,16740,18299,-,narH,Op473,Energy Production & Conversion,NZ_JAJGVJ010000039.1, LMB90_RS01290,0.2917620160099222,18289,21954,-,LMB90_RS01290,Op509,Energy Production & Conversion,NZ_JAJGVJ010000039.1,
LMB90_RS01295,0.1092855830677148,22099,23118,LMB90_RS01295,Op589,Coenzyme Transport & Metabolism,NZ_JAJGVJ010000039.1, LMB90_RS01300,0.0819915851630387,23115,23612,LMB90_RS01300,Op615,Coenzyme Transport & Metabolism,NZ_JAJGVJ010000039.1,
LMB90_RS01305,0.1073732866463989,23602,24819,LMB90_RS01305,Op626,Coenzyme Transport & Metabolism,NZ_JAJGVJ010000039.1, LMB90_RS01310,0.0816776320282681,24798,25289,mobB,Op648,Coenzyme Transport & Metabolism,NZ_JAJGVJ010000039.1, LMB90_RS01315,0.0584347241689387,25286,25858,LMB90_RS01315,Op661,Coenzyme Transport & Metabolism,NZ_JAJGVJ010000039.1,
LMB90_RS01320,0.1055358106494458,25848,26132,LMB90_RS01320,Op671,No COG Annotation,NZ_JAJGVJ010000039.1,
LMB90_RS01335,0.1349089263577249,28084,28356,LMB90_RS01335,Op707,No COG Annotation,NZ_JAJGVJ010000039.1,
LMB90_RS01340,0.1757467843962169,28362,29330,LMB90_RS01340,Op715,No COG Annotation,NZ_JAJGVJ010000039.1,
LMB90_RS01345,0.1056614949312385,29335,30243,LMB90_RS01345,Op737,Coenzyme Transport & Metabolism,NZ_JAJGVJ010000039.1, LMB90_RS01350,0.2109399828908563,30303,31496,-,LMB90_RS01350,Op756,Inorganic Ion Transport & Metabolism,NZ_JAJGVJ010000039.1,
LMB90_RS01355,0.2722092774504661,31548,31793,-,LMB90_RS01355,Op783,Coenzyme Transport & Metabolism,NZ_JAJGVJ010000039.1, LMB90_RS01360,0.2670582038416618,31793,32149,-,LMB90_RS01360,Op789,Coenzyme Transport & Metabolism,NZ_JAJGVJ010000039.1,
LMB90_RS01385,0.2508166293348772,35741,36700,LMB90_RS01385,Op870,No COG Annotation,NZ_JAJGVJ010000039.1,
LMB90_RS01390,0.2537308638745905,36716,37147,LMB90_RS01390,Op893,No COG Annotation,NZ_JAJGVJ010000039.1,
LMB90_RS05330,0.1386534845486849,14779,16020,LMB90_RS05330,Op422,Amino Acid Transport & Conversion,NZ_JAJGVJ010000058.1, LMB90_RS05335,0.1383463163025766,16013,17311,preA,Op453,Energy Production & Conversion,NZ_JAJGVJ010000058.1,

ChatGPT:

Name: Nitrate Reductase and Cofactor Metabolism iModulon

Biological Function: The 'Nitrate Reduction and Cofactor Metabolism' iModulon likely plays a role in nitrate reduction processes and the metabolism of essential cofactors, particularly molybdenum (Mo) and potentially menaquinone (Vitamin K2). This iModulon incorporates genes associated with several key functions:

1. **Nitrate Reduction**: The presence of the NAR operon genes (narI, narJ, narH) suggests a primary role in nitrate reduction. Nitrate reductase requires molybdenum (Mo) as a cofactor for its physiological operation, and the genes in this operon may be involved in the conversion of nitrate to nitrite.

2. **Molybdenum Cofactor Metabolism**: The gene mobB, a molybdenum cofactor gene, is included in this iModulon. This indicates a potential role in the biosynthesis or regulation of molybdenum cofactors essential for the activity of molybdoenzymes, including nitrate reductase.

3. **Coenzyme Transport & Metabolism**: Several genes associated with coenzyme transport and metabolism (LMB90_RS01345, LMB90_RS01355, LMB90_RS01360, LMB90_RS01385, LMB90_RS01390) are present. These genes may be involved in the transport and metabolism of cofactors required for various enzymatic reactions.

4. **Inorganic Ion Transport & Metabolism**: The gene LMB90_RS01350, associated with inorganic ion transport and metabolism, may have a role in the transport of ions or cofactors involved in nitrate reduction or other metabolic processes.

5. **Amino Acid Transport & Conversion**: Genes related to amino acid transport and conversion (LMB90_RS01270, LMB90_RS05330) may be associated with the uptake and utilization of amino acids or their role in nitrogen metabolism.

6. **Energy Production & Conversion**: Genes associated with energy production and conversion (LMB90_RS01275, LMB90_RS01280, LMB90_RS01285, LMB90_RS05335) are likely involved in energy generation processes associated with nitrate reduction and metabolic reactions.

7. **Coenzyme Regulation**: Genes related to coenzyme metabolism (LMB90_RS01395, LMB90_RS01300, LMB90_RS01305, LMB90_RS01310, LMB90_RS01315, LMB90_RS01320) may play a role in regulating coenzyme levels, ensuring proper cofactor availability for metabolic reactions.

8. **Vitamin K2 Metabolism (Potential)**: The gene preA is associated with energy production and conversion. It may also be involved in menaquinone (Vitamin K2) biosynthesis, although further experimental evidence is needed to confirm this.

This iModulon likely functions in coordinating the metabolism of cofactors like molybdenum and the reduction of nitrate, which can serve as an alternative electron acceptor in anaerobic respiration. Additionally, its association with coenzyme metabolism suggests a role in maintaining proper cofactor levels for various cellular processes. Further experimental studies would be necessary to validate its specific functions and regulatory mechanisms.

Uncharacterized iModulon 10
Jonathan Josephs-Spaulding:
Given the following information, I would like to characterize 'uncharacterized iModulon 10'. Please suggest a name to describe this iModulon and a potential biological function. However, if you cannot make a decision due to limited information, please state that this iModulon is ‘Uncharacterized’:
,gene_weight,start,end,strand,gene_name,COG,accession,regulator
LMB90_RS00540,0.1986547483115125,21338,21658,LMB90_RS00540,Op574,Function Unknown,NZ_JAJGVJ010000030.1, LMB90_RS01960,0.1163766892605224,67469,68443,-,LMB90_RS01960,Op1386,Energy Production & Conversion,NZ_JAJGVJ010000045.1, LMB90_RS01965,0.1283478746017215,68471,69211,-,LMB90_RS01965,Op1402,Cell Wall/Membrane/Envelope Biogenesis,NZ_JAJGVJ010000045.1,CcpA LMB90_RS01970,0.1378564090360538,69204,69623,-,LMB90_RS01970,Op1411,Function Unknown,NZ_JAJGVJ010000045.1,CcpA LMB90_RS02120,0.0781341174837284,99602,99985,-,LMB90_RS02120,Op1752,No COG Annotation,NZ_JAJGVJ010000045.1, LMB90_RS02335,0.1325039766007641,25020,25883,LMB90_RS02335,Op654,"Intracellular Trafficking, Secretion, & Vesicular Transport",NZ_JAJGVJ010000046.1, LMB90_RS02350,-0.150520219712584,27889,29262,LMB90_RS02350,Op703,Energy Production & Conversion,NZ_JAJGVJ010000046.1,CcpA LMB90_RS02515,0.1916475983608627,75339,76688,LMB90_RS02515,Op1501,Energy Production & Conversion,NZ_JAJGVJ010000046.1,Rex LMB90_RS02630,-0.0800004849078802,17937,19616,-,alsS,Op499,Amino Acid Transport & Conversion,NZ_JAJGVJ010000047.1, LMB90_RS02775,0.1426359725847653,5310,6698,LMB90_RS02775,Op165,Energy Production & Conversion,NZ_JAJGVJ010000050.1, LMB90_RS02780,0.1252314844180106,6820,8214,LMB90_RS02780,Op201,Energy Production & Conversion,NZ_JAJGVJ010000050.1, LMB90_RS02785,0.0897246449845746,8287,9708,LMB90_RS02785,Op244,Energy Production & Conversion,NZ_JAJGVJ010000050.1, LMB90_RS02980,0.2030107259663709,42137,43165,adhP,Op987,Energy Production & Conversion,NZ_JAJGVJ010000050.1, LMB90_RS04065,0.294182218376938,42267,44903,adhE,Op990,Energy Production & Conversion,NZ_JAJGVJ010000052.1,"CcpA,Rex" LMB90_RS04435,0.1075800618930559,54123,55046,rbsK,Op1185,Coenzyme

Transport & Metabolism,NZ_JAJGVJ010000055.1,"CcpA,CcpA"
LMB90_RS04440,0.0794544251011108,55058,55453,rbsD,Op1199,Carbohydrate Transport & Metabolism,NZ_JAJGVJ010000055.1,"CcpA,CcpA"
LMB90_RS04445,0.0812242835881021,55473,56828,fucP,Op1207,Carbohydrate Transport & Metabolism,NZ_JAJGVJ010000055.1,CcpA LMB90_RS04645,0.08773978002744,104745,105221,LMB90_RS04645,Op1784,No COG Annotation,NZ_JAJGVJ010000055.1, LMB90_RS05330,0.1562682544190294,14779,16020,LMB90_RS05330,Op422,Amino Acid Transport & Conversion,NZ_JAJGVJ010000058.1, LMB90_RS05335,0.1321968221066291,16013,17311,preA,Op453,Energy Production & Conversion,NZ_JAJGVJ010000058.1,
LMB90_RS05835,-0.0958824228984198,7474,7785,-,LMB90_RS05835,Op221,"Intracellular Trafficking, Secretion, & Vesicular Transport",NZ_JAJGVJ010000059.1, LMB90_RS06155,0.1642312458916394,82266,83009,LMB90_RS06155,Op1586,Function Unknown,NZ_JAJGVJ010000059.1, LMB90_RS06160,0.1670975520062348,83034,83441,LMB90_RS06160,Op1597,Amino Acid Transport & Conversion,NZ_JAJGVJ010000059.1,
LMB90_RS06660,-0.1494356183495593,72457,73212,LMB90_RS06660,Op1462,"Replication, Recombination, & Repair",NZ_JAJGVJ010000060.1, LMB90_RS06665,-0.1364756271154591,73202,73492,LMB90_RS06665,Op1474,"Replication, Recombination, & Repair",NZ_JAJGVJ010000060.1,
LMB90_RS06670,-0.1321794235469896,73494,74486,LMB90_RS06670,Op1479,Energy Production & Conversion,NZ_JAJGVJ010000060.1, LMB90_RS06930,0.0761170025597312,432,1868,-,gndA,Op40,No COG Annotation,NZ_JAJGVJ010000062.1, LMB90_RS08635,0.1373710887651493,45436,46446,LMB90_RS08635,Op1050,No COG Annotation,NZ_JAJGVJ010000066.1, LMB90_RS08735,0.1246168173216513,66133,67254,-,LMB90_RS08735,Op1366,Cell Wall/Membrane/Envelope Biogenesis,NZ_JAJGVJ010000066.1,Rex
LMB90_RS08870,0.0762168172685661,94523,95551,-,LMB90_RS08870,Op1707,"Intracellular Trafficking, Secretion, & Vesicular Transport",NZ_JAJGVJ010000066.1,
ChatGPT:
Name: "Sugar Fermentation Regulator Modulon"

Potential Biological Function: The genes in this iModulon appear to be primarily involved in various aspects of energy metabolism from sugars. This includes genes associated with energy production and conversion, coenzyme transport and metabolism, and amino acid transport and conversion. Some of the genes are regulated by CcpA and Rex, which are known to be involved in the control of energy-related processes.

Therefore, the potential biological function of 'uncharacterized iModulon 10' could be related to the regulation of energy metabolism in the organism. This may involve the utilization of different energy sources, coenzyme metabolism, and adaptation to changing energy conditions. Further experimental studies would be needed to validate this hypothesis and uncover the specific role of this iModulon in the organism's physiology.

Uncharacterized iModulon 11
Jonathan Josephs-Spaulding:
Given the following information, I would like to characterize 'uncharacterized iModulon 11'. Please suggest a name to describe this iModulon and a potential biological function. However, if you cannot make a decision due to limited information, please state that this iModulon is 'Uncharacterized': ,gene_weight,start,end,strand,gene_name,COG,accession,regulator
LMB90_RS00950,0.077085838759445,117300,118676,LMB90_RS00950,Op1868,"Intracellular Trafficking, Secretion, & Vesicular Transport",NZ_JAJGVJ010000030.1,PyrR
LMB90_RS01315,-0.0555229778730122,25286,25858,LMB90_RS01315,Op661,Coenzyme Transport & Metabolism,NZ_JAJGVJ010000039.1, LMB90_RS01335,-0.1281355887301037,28084,28356,LMB90_RS01335,Op707,No COG Annotation,NZ_JAJGVJ010000039.1, LMB90_RS01635,0.058189045029759,3006,3371,-,LMB90_RS01635,Op104,Function Unknown,NZ_JAJGVJ010000045.1, LMB90_RS01670,0.1001685897031578,6840,7478,-,LMB90_RS01670,Op202,Function Unknown,NZ_JAJGVJ010000045.1, LMB90_RS01675,0.1945426828483145,7488,7931,-,LMB90_RS01675,Op223,Function Unknown,NZ_JAJGVJ010000045.1, LMB90_RS01700,0.0825336101342366,13503,14279,-,LMB90_RS01700,Op392,No COG Annotation,NZ_JAJGVJ010000045.1, LMB90_RS02465,0.065532693532263,65010,65441,LMB90_RS02465,Op1348,No COG Annotation,NZ_JAJGVJ010000046.1, LMB90_RS02655,0.0896415211104715,331,579,-,LMB90_RS02655,Op36,Function Unknown,NZ_JAJGVJ010000048.1, LMB90_RS02765,0.0554304047357559,2370,3251,-,LMB90_RS02765,Op84,Carbohydrate Transport & Metabolism,NZ_JAJGVJ010000050.1,MleR
LMB90_RS02805,0.0569297389345105,12928,14487,LMB90_RS02805,Op376,"Replication, Recombination, & Repair",NZ_JAJGVJ010000050.1, LMB90_RS03535,0.0935645169378068,55759,56331,LMB90_RS03535,Op1213,Function Unknown,NZ_JAJGVJ010000051.1,BirA LMB90_RS04045,0.0865942172825067,37498,38010,-,tadA,Op906,Nucleotide Transport & Metabolism,NZ_JAJGVJ010000052.1, LMB90_RS04055,-0.0570014322975744,38589,40760,nrdE,Op930,Nucleotide Transport & Metabolism,NZ_JAJGVJ010000052.1,NrdR
LMB90_RS04085,0.1250366263179479,2574,3104,-,LMB90_RS04085,Op90,Function Unknown,NZ_JAJGVJ010000053.1,
LMB90_RS04150,0.0895626693865859,14182,15111,LMB90_RS04150,Op408,Function Unknown,NZ_JAJGVJ010000053.1,
LMB90_RS04195,0.0634175919628529,1770,3104,LMB90_RS04195,Op69,"Replication, Recombination, & Repair",NZ_JAJGVJ010000055.1, LMB90_RS04580,0.0577126420154472,88122,89630,xylB,Op1648,Carbohydrate Transport &

Metabolism,NZ_JAJGVJ010000055.1,
LMB90_RS04605,0.0900173865556575,96958,97797,-,LMB90_RS04605,Op1728,Transcription,NZ_JAJGVJ010000055.1,
LMB90_RS04845,0.1688740500813501,3891,4721,LMB90_RS04845,Op128,Function Unknown,NZ_JAJGVJ010000056.1,
LMB90_RS05430,0.1001089412754497,35859,36905,LMB90_RS05430,Op872,Cell Motility,NZ_JAJGVJ010000058.1,
LMB90_RS05825,0.1201395819451503,6766,7056,-,LMB90_RS05825,Op196,No COG Annotation,NZ_JAJGVJ010000059.1,
LMB90_RS05835,0.2662640462765817,7474,7785,-,LMB90_RS05835,Op221,"Intracellular Trafficking, Secretion, & Vesicular Transport",NZ_JAJGVJ010000059.1, LMB90_RS05840,0.1470126026934256,7785,8855,-,LMB90_RS05840,Op231,Cell Motility,NZ_JAJGVJ010000059.1, LMB90_RS05845,0.0953188031671372,8773,9750,-,tadA,Op257,Cell Motility,NZ_JAJGVJ010000059.1, LMB90_RS06200,0.1441947766785059,89018,89587,-,LMB90_RS06200,Op1655,Function Unknown,NZ_JAJGVJ010000059.1, LMB90_RS06425,0.197465753664903,30914,31546,LMB90_RS06425,Op766,"Replication, Recombination, & Repair",NZ_JAJGVJ010000060.1,
LMB90_RS07010,0.0801114909462376,16135,17877,LMB90_RS07010,Op456,"Intracellular Trafficking, Secretion, & Vesicular Transport",NZ_JAJGVJ010000062.1, LMB90_RS07030,0.0693472958487749,21914,22645,LMB90_RS07030,Op583,Amino Acid Transport & Conversion,NZ_JAJGVJ010000062.1,
LMB90_RS07110,0.0885609577222035,39413,40294,-,LMB90_RS07110,Op938,Lipid Transport & Metabolism,NZ_JAJGVJ010000062.1, LMB90_RS07230,0.1521338077331263,65365,66213,LMB90_RS07230,Op1355,No COG Annotation,NZ_JAJGVJ010000062.1,
LMB90_RS07460,0.0559594388593206,119649,120494,-,LMB90_RS07460,Op1880,Function Unknown,NZ_JAJGVJ010000062.1,
LMB90_RS07650,0.0549417851326189,73448,74386,LMB90_RS07650,Op1478,Nucleotide Transport & Metabolism,NZ_JAJGVJ010000063.1, LMB90_RS07895,0.0819585862438841,31800,32450,LMB90_RS07895,Op790,Function Unknown,NZ_JAJGVJ010000064.1, LMB90_RS08045,0.118330772989309,59449,61371,LMB90_RS08045,Op1266,No COG Annotation,NZ_JAJGVJ010000064.1,"CcpA,LacR"
LMB90_RS08050,0.0949905037943816,61381,63399,LMB90_RS08050,Op1295,Transcription,NZ_JAJGVJ010000064.1,"CcpA,LacR" LMB90_RS08070,0.0556404863636331,66500,67639,-,LMB90_RS08070,Op1373,Coenzyme Transport & Metabolism,NZ_JAJGVJ010000064.1,T-box(Met) LMB90_RS08255,0.0823467966119702,24515,25360,rfbD,Op644,Cell Wall/Membrane/Envelope Biogenesis,NZ_JAJGVJ010000065.1,
LMB90_RS08390,0.0739946114412486,59258,59818,LMB90_RS08390,Op1264,Nucleotide Transport & Metabolism,NZ_JAJGVJ010000065.1,
LMB90_RS08475,0.0604227121353243,10648,11292,LMB90_RS08475,Op311,Transcription,NZ_JAJGVJ010000066.1,
LMB90_RS08495,0.0708969339495442,13523,14854,-,LMB90_RS08495,Op393,Nucleotide Transport & Metabolism,NZ_JAJGVJ010000066.1, LMB90_RS08500,0.0570832742863857,14844,15341,-,LMB90_RS08500,Op424,Signal Transduction Mechanisms,NZ_JAJGVJ010000066.1,
LMB90_RS08575,0.1982319097851601,30941,31378,LMB90_RS08575,Op767,Transcription,NZ_JAJGVJ010000066.1,
LMB90_RS08660,0.0632219092815737,51129,51365,LMB90_RS08660,Op1143,Defense Mechanisms,NZ_JAJGVJ010000066.1,
LMB90_RS08840,0.0539978908380299,88795,90117,-,LMB90_RS08840,Op1654,Inorganic Ion Transport & Metabolism,NZ_JAJGVJ010000066.1,
LMB90_RS09025,-0.0628975062532757,123595,124098,LMB90_RS09025,Op1895,"Posttranslational Modifications, Protein Turnover, Chaperones",NZ_JAJGVJ010000066.1,
LMB90_RS09030,0.0782080815440354,124199,125359,LMB90_RS09030,Op1897,Transcription,NZ_JAJGVJ010000066.1,
LMB90_RS09155,0.0697917860676322,148633,149739,LMB90_RS09155,Op1946,Transcription,NZ_JAJGVJ010000066.1,
LMB90_RS09410,0.0532486861910577,46469,47239,-,LMB90_RS09410,Op1065,"Translation, Ribosomal Structure, & Biogenesis",NZ_JAJGVJ010000067.1, LMB90_RS09430,0.0754788721374177,50413,51090,-,LMB90_RS09430,Op1129,No COG Annotation,NZ_JAJGVJ010000067.1, LMB90_RS09475,0.0770099068915123,12527,13420,LMB90_RS09475,Op364,Signal Transduction Mechanisms,NZ_JAJGVJ010000068.1,
LMB90_RS09580,0.0811668124972526,29258,30157,pstC,Op733,Transcription,NZ_JAJGVJ010000068.1,
LMB90_RS10115,0.0530184271091172,65014,65943,LMB90_RS10115,Op1349,Cell Wall/Membrane/Envelope Biogenesis,NZ_JAJGVJ010000069.1,

ChatGPT:
Name: Intestinal Carbohydrate and Host Interaction iModulon

Biological Function: The 'Intestinal Carbohydrate and Host Interaction' iModulon likely plays a role in mediating the interaction between the bacterium and its host in the intestinal environment. This iModulon incorporates genes associated with various functions:

1. **Pili Formation and Biofilm Formation**: The presence of tadA genes suggests involvement in forming pili, which are important for mediating bacterial attachment to surfaces and biofilm formation. In the context of the intestine, this may be related to bacterial attachment to the gastrointestinal (GI) tract, potentially influencing host colonization.

2. **Ribonucleotide Reductase Activity**: The nrdE gene is involved in ribonucleotide reductase activity, which is crucial for synthesizing deoxyribonucleotides needed for DNA replication and genome stability.

3. **Xylose Sugar Uptake**: The gene xylB is associated with the uptake of xylose sugars, potentially related to the utilization of dietary xylose in the intestine.

4. **Biosynthesis of Molecules and Antigens**: The rfbD gene is involved in the biosynthesis of molecules and antigens that may interact with the host immune system. This suggests a role in modulating the host-bacterial immune response.

5. **Phosphate Metabolism**: The pstC gene is associated with phosphate metabolism, which could influence nutrient acquisition and utilization in the intestine.

6. **Regulation by Various Factors**: Predicted regulators of this iModulon include PyrR (associated with pyrimidine biosynthesis), MelR (related to melibiose utilization), BirA (involved in biotin biosynthesis), NrdR (regulating ribonucleotide synthesis), LacR (associated with lactose metabolism), and CcpA (carbon catabolite control). These regulators suggest that the iModulon responds to different dietary and metabolic signals.

The 'Intestinal Carbohydrate and Host Interaction' iModulon likely plays a multifaceted role in mediating bacterial attachment, utilizing dietary sugars, influencing the host's immune response, and responding to various regulatory signals related to nutrient availability and metabolism in the intestine. This iModulon may be more highly expressed in response to plant-based diets or in the presence of specific dietary components in the intestinal environment. Further experimental studies are needed to validate its specific functions and regulatory mechanisms.

Uncharacterized iModulon 15
Jonathan Josephs-Spaulding:
Given the following information, I would like to characterize 'uncharacterized iModulon 15'. Please suggest a name to describe this iModulon and a potential biological function. However, if you cannot make a decision due to limited information, please state that this iModulon is 'Uncharacterized':
,gene_weight,start,end,strand,gene_name,COG,accession,regulator
LMB90_RS01325,0.2711945173737334,26283,27341,LMB90_RS01325,Op680,Nucleotide Transport & Metabolism,NZ_JAJGVJ010000039.1,
LMB90_RS01330,0.2551482889338795,27334,27987,LMB90_RS01330,Op694,Transcription,NZ_JAJGVJ010000039.1,
LMB90_RS02010,0.083968113428236,77162,78052,-,LMB90_RS02010,Op1525,Transcription,NZ_JAJGVJ010000045.1,
LMB90_RS02015,0.1059225378436966,78069,78401,-,LMB90_RS02015,Op1536,Function Unknown,NZ_JAJGVJ010000045.1,
LMB90_RS02020,0.1182200011262587,78434,79843,-,sufB,Op1542,"Posttranslational Modifications, Protein Turnover, Chaperones",NZ_JAJGVJ010000045.1,
LMB90_RS02025,0.125676877290463,79836,80297,-,LMB90_RS02025,Op1559,Energy Production & Conversion,NZ_JAJGVJ010000045.1,
LMB90_RS02030,0.1257223101396903,80284,81522,-,LMB90_RS02030,Op1563,Amino Acid Transport & Conversion,NZ_JAJGVJ010000045.1,
LMB90_RS02035,0.1324120270770544,81503,82792,-,sufD,Op1581,"Posttranslational Modifications, Protein Turnover, Chaperones",NZ_JAJGVJ010000045.1,
LMB90_RS02040,0.1403587317552039,82804,83601,-,sufC,Op1595,"Posttranslational Modifications, Protein Turnover, Chaperones",NZ_JAJGVJ010000045.1,
LMB90_RS02045,0.1961501547505179,83836,84042,LMB90_RS02045,Op1603,Inorganic Ion Transport & Metabolism,NZ_JAJGVJ010000045.1,
LMB90_RS02050,0.1742365285910009,84044,86035,feoB,Op1606,Inorganic Ion Transport & Metabolism,NZ_JAJGVJ010000045.1,
LMB90_RS02055,0.17922483777878,86042,86200,LMB90_RS02055,Op1626,No COG Annotation,NZ_JAJGVJ010000045.1,
LMB90_RS02510,-0.0851520490891521,74573,75070,-,LMB90_RS02510,Op1489,Function Unknown,NZ_JAJGVJ010000046.1,
LMB90_RS02515,0.1585503164764941,75339,76688,LMB90_RS02515,Op1501,Energy Production & Conversion,NZ_JAJGVJ010000046.1,Rex
LMB90_RS05330,-0.2035581438428592,14779,16020,LMB90_RS05330,Op422,Amino Acid Transport & Conversion,NZ_JAJGVJ010000058.1,
LMB90_RS05335,-0.2034132883676374,16013,17311,preA,Op453,Energy Production & Conversion,NZ_JAJGVJ010000058.1,
LMB90_RS09080,-0.0881283480086439,135396,136148,-,pnuC,Op1927,Transcription,NZ_JAJGVJ010000066.1,NrdR

ChatGPT:
Name: I would suggest naming this iModulon "Fe-S Cluster Metabolism Modulon" because it includes genes associated with iron and sulfur metabolism, as well as some genes related to transcription.

Potential Biological Function: This iModulon appears to be involved in the metabolism of iron and sulfur, which are essential elements in various cellular processes. Specifically, it contains genes associated with the Suf operon (sufB, sufD, sufC), which is involved in iron-sulfur cluster assembly, a crucial process for various metalloenzymes.

Additionally, it includes genes related to iron transport (feoB) and genes associated with transcriptional regulation (LMB90_RS02010, LMB90_RS02015, LMB90_RS09080). The presence of transcription-related genes suggests a potential regulatory role for this iModulon in coordinating the expression of genes involved in iron and sulfur metabolism.

The negative gene weights for LMB90_RS02510, LMB90_RS05330, LMB90_RS05335, and LMB90_RS09080 indicate potential regulatory interactions where these genes might act as repressors or have inhibitory effects on certain cellular processes.

Overall, this iModulon likely plays a role in iron and sulfur metabolism and may be involved in coordinating the cellular response to iron and sulfur availability. However, further experimental studies would be needed to confirm its exact biological function and regulatory mechanisms.

Uncharacterized iModulon 16
Jonathan Josephs-Spaulding:
Given the following information, I would like to characterize 'uncharacterized iModulon 16'. Please suggest a name to describe this iModulon and a potential biological function. However, if you cannot make a decision due to limited information, please state that this iModulon is 'Uncharacterized':
,gene_weight,start,end,strand,gene_name,COG,accession,regulator
LMB90_RS01145,0.0986142217356553,1,246,-,LMB90_RS01145,Op16,No COG Annotation,NZ_JAJGVJ010000038.1,
LMB90_RS01870,0.0961881228641606,47577,48053,-,LMB90_RS01870,Op1085,Nucleotide Transport & Metabolism,NZ_JAJGVJ010000045.1,
LMB90_RS03610,0.1596178824763088,71777,72019,-,LMB90_RS03610,Op1451,Function Unknown,NZ_JAJGVJ010000051.1,
LMB90_RS05085,0.0882366420278246,50867,51097,-,LMB90_RS05085,Op1137,No COG Annotation,NZ_JAJGVJ010000056.1,
LMB90_RS05140,0.1191220218663702,7126,7302,LMB90_RS05140,Op209,Function Unknown,NZ_JAJGVJ010000057.1,
LMB90_RS05360,0.0948234346101933,21045,21569,-,LMB90_RS05360,Op565,Function Unknown,NZ_JAJGVJ010000058.1,
LMB90_RS06165,0.1276566272107964,83508,83780,-,LMB90_RS06165,Op1600,No COG Annotation,NZ_JAJGVJ010000059.1,
LMB90_RS07475,0.1125667644420368,116,520,-,LMB90_RS07475,Op26,No COG Annotation,NZ_JAJGVJ010000063.1,
LMB90_RS07775,0.0891224007255211,9580,9861,rpmA,Op281,"Translation, Ribosomal Structure, & Biogenesis",NZ_JAJGVJ010000064.1,
LMB90_RS07900,0.1056573368928294,32600,32785,rpmB,Op810,Carbohydrate Transport & Metabolism,NZ_JAJGVJ010000064.1,
LMB90_RS08165,0.1388298834751388,8784,9020,-,LMB90_RS08165,Op258,Nucleotide Transport & Metabolism,NZ_JAJGVJ010000065.1,
LMB90_RS09230,0.1044762253511485,13028,13162,rpmH,Op380,"Replication, Recombination, & Repair",NZ_JAJGVJ010000067.1,

ChatGPT:
Based on the provided information, we can characterize 'uncharacterized iModulon 16' as follows:

Name: I would suggest naming this iModulon "Ribosomal Protein Modulon" because it includes genes associated with ribosomal proteins (rpmA, rpmB, rpmH), which are essential components of ribosomes, the cellular machinery responsible for protein synthesis.

Potential Biological Function: This iModulon appears to be involved in regulating the expression of ribosomal proteins. Ribosomal proteins are essential for the formation and function of ribosomes, which are responsible for protein synthesis in cells. The presence of genes related to ribosomal proteins in this iModulon suggests that it may play a role in coordinating the expression of ribosomal components in response to cellular needs for protein synthesis.

Ribosomal proteins are often regulated to match the demands of the cell for protein production. Therefore, this iModulon could be involved in modulating the cellular protein synthesis machinery to adapt to changing environmental conditions or growth phases.

Additionally, some other genes with functions related to translation, nucleotide transport, and replication are present in this iModulon, suggesting a broader role in cellular processes related to protein synthesis and nucleotide metabolism.

Overall, this iModulon likely plays a role in regulating the expression of ribosomal proteins and may be involved in coordinating protein synthesis in response to cellular needs. However, further experimental studies would be needed to confirm its exact biological function and regulatory mechanisms.

Uncharacterized iModulon 17
Jonathan Josephs-Spaulding:
Given the following information, I would like to characterize 'uncharacterized iModulon 17'. Please suggest a name to describe this iModulon and a potential biological function. However, if you cannot make a decision due to limited information, please state that this iModulon is 'Uncharacterized':
,gene_weight,start,end,strand,gene_name,COG,accession,regulator
LMB90_RS01960,0.2268572590392429,67469,68443,-,LMB90_RS01960,Op1386,Energy Production & Conversion,NZ_JAJGVJ010000045.1,
LMB90_RS01965,0.2213033877203007,68471,69211,-,LMB90_RS01965,Op1402,Cell Wall/Membrane/Envelope Biogenesis,NZ_JAJGVJ010000045.1,CcpA
LMB90_RS01970,0.2412036308460802,69204,69623,-,LMB90_RS01970,Op1411,Function Unknown,NZ_JAJGVJ010000045.1,CcpA
LMB90_RS02120,-0.1471500288470342,99602,99985,-,LMB90_RS02120,Op1752,No COG Annotation,NZ_JAJGVJ010000045.1,
LMB90_RS07165,-0.1356325059215536,51367,52020,-,LMB90_RS07165,Op1148,Inorganic Ion Transport & Metabolism,NZ_JAJGVJ010000062.1,TPP Riboswitch

ChatGPT:
Based on the provided information, we can characterize 'uncharacterized iModulon 17' as follows:

Name: Uncharacterized-1

The diverse functions of these genes suggest that this iModulon may play a role in coordinating multiple aspects of cellular regulation. However, the precise biological function of this iModulon cannot be definitively determined due to its heterogeneity and the limited available information. As such, this iModulon is categorized as 'Uncharacterized.' Further experimental studies would be necessary to elucidate its specific role within the cellular regulatory network.

Uncharacterized iModulon 20
Jonathan Josephs-Spaulding:
Given the following information, I would like to characterize 'uncharacterized iModulon 20'. Please suggest a name to describe this iModulon and a potential biological function. However, if you cannot make a decision due to limited information, please state that this iModulon is 'Uncharacterized': ,gene_weight,start,end,strand,gene_name,COG,accession,regulator
LMB90_RS00460,0.1236610908192633,4480,5130,LMB90_RS00460,Op146,Function Unknown,NZ_JAJGVJ010000030.1,
LMB90_RS00535,0.1274490611833283,19696,21051,-,LMB90_RS00535,Op538,Energy Production & Conversion,NZ_JAJGVJ010000030.1, LMB90_RS00595,0.0908625094030802,31184,31612,msrB,Op773,"Posttranslational Modifications, Protein Turnover, Chaperones",NZ_JAJGVJ010000030.1,
LMB90_RS01700,-0.075153021178907,13503,14279,-,LMB90_RS01700,Op392,No COG Annotation,NZ_JAJGVJ010000045.1,
LMB90_RS02235,0.0730355119554146,5578,6780,LMB90_RS02235,Op172,Amino Acid Transport & Conversion,NZ_JAJGVJ010000046.1, LMB90_RS02240,0.0721918739814814,6793,7119,LMB90_RS02240,Op198,No COG Annotation,NZ_JAJGVJ010000046.1, LMB90_RS02350,0.0804000832983706,27889,29262,LMB90_RS02350,Op703,Energy Production & Conversion,NZ_JAJGVJ010000046.1,CcpA
LMB90_RS02390,0.1052498934262734,37950,38378,-,LMB90_RS02390,Op914,"Posttranslational Modifications, Protein Turnover, Chaperones",NZ_JAJGVJ010000046.1, LMB90_RS02735,0.0799602615696568,3272,3910,-,LMB90_RS02735,Op110,"Secondary Metabolites Biosynthesis, Transport, & Catabolism",NZ_JAJGVJ010000049.1,
LMB90_RS03795,0.0993656780411451,112204,112608,LMB90_RS03795,Op1832,Function Unknown,NZ_JAJGVJ010000051.1,
LMB90_RS04265,0.0919810658850166,16111,17043,trxB,Op455,Energy Production & Conversion,NZ_JAJGVJ010000055.1,
LMB90_RS05345,0.0917954274297253,18534,18965,LMB90_RS05345,Op515,"Posttranslational Modifications, Protein Turnover, Chaperones",NZ_JAJGVJ010000058.1, LMB90_RS05625,0.1034094174988329,72171,72491,LMB90_RS05625,Op1456,Energy Production & Conversion,NZ_JAJGVJ010000058.1,
LMB90_RS05915,0.0828072505379885,19851,20165,-,trxA,Op543,"Posttranslational Modifications, Protein Turnover, Chaperones",NZ_JAJGVJ010000059.1, LMB90_RS06140,0.1097490078439748,78094,79425,-,frc,Op1539,Energy Production & Conversion,NZ_JAJGVJ010000059.1, LMB90_RS06145,0.1200823385518608,79418,81154,-,oxc,Op1556,Amino Acid Transport & Conversion,NZ_JAJGVJ010000059.1, LMB90_RS06165,0.0818982657030855,83508,83780,-,LMB90_RS06165,Op1600,No COG Annotation,NZ_JAJGVJ010000059.1, LMB90_RS07120,0.0815566835966715,41793,43205,LMB90_RS07120,Op981,Defense

Mechanisms,NZ_JAJGVJ010000062.1, LMB90_RS07185,0.0831059559934193,55070,55825,-,LMB90_RS07185,Op1200,Function Unknown,NZ_JAJGVJ010000062.1, LMB90_RS07290,0.0692770793302669,80705,81997,LMB90_RS07290,Op1572,Inorganic Ion Transport & Metabolism,NZ_JAJGVJ010000062.1,
LMB90_RS08020,0.0699928159994093,56035,56259,-,LMB90_RS08020,Op1218,No COG Annotation,NZ_JAJGVJ010000064.1,
LMB90_RS08535,0.1054915035112392,23639,24280,-,LMB90_RS08535,Op628,Function Unknown,NZ_JAJGVJ010000066.1,
LMB90_RS08540,0.0735454399336075,24485,25807,LMB90_RS08540,Op643,Amino Acid Transport & Conversion,NZ_JAJGVJ010000066.1, LMB90_RS08560,0.1067127788896077,28967,29569,-,LMB90_RS08560,Op729,No COG Annotation,NZ_JAJGVJ010000066.1, LMB90_RS08700,0.0704881085968185,59115,59609,-,LMB90_RS08700,Op1262,Amino Acid Transport & Conversion,NZ_JAJGVJ010000066.1,
LMB90_RS09285,0.1097059510267585,25645,26508,LMB90_RS09285,Op667,"Intracellular Trafficking, Secretion, & Vesicular Transport",NZ_JAJGVJ010000067.1, LMB90_RS09380,0.0733688980889804,42670,43347,-,LMB90_RS09380,Op1000,Defense Mechanisms,NZ_JAJGVJ010000067.1,
LMB90_RS09495,0.0714781971508564,16344,16739,LMB90_RS09495,Op463,Transcription,NZ_JAJGVJ010000068.1,
LMB90_RS09725,0.0849819781316903,64955,65395,LMB90_RS09725,Op1346,Defense Mechanisms,NZ_JAJGVJ010000068.1,
LMB90_RS09920,0.0865943585486321,33585,34916,LMB90_RS09920,Op831,Function Unknown,NZ_JAJGVJ010000069.1,
LMB90_RS10045,0.0727592957299813,54189,54428,-,LMB90_RS10045,Op1186,"Replication, Recombination, & Repair",NZ_JAJGVJ010000069.1,

ChatGPT:

Based on the provided information, we can characterize 'uncharacterized iModulon 20' as follows:

Name: I would suggest naming this iModulon "Oxidative Stress Response Modulon" because it contains genes associated with various functions, including stress response, energy production, and protein turnover.

Potential Biological Function: This iModulon appears to play a role in adapting to stress conditions and metabolic adjustments within the cell.

1. **Stress Response:** Several genes in this iModulon, such as LMB90_RS02735, LMB90_RS07120, LMB90_RS09380, and LMB90_RS09725, are associated with defense mechanisms and stress response. These genes may be involved in the cell's response to environmental stressors and protection against harmful agents.

2. **Energy Production & Conversion:** Genes like LMB90_RS00535, LMB90_RS02350, LMB90_RS04265, and LMB90_RS05625 are involved in energy production and conversion. They are crucial for maintaining cellular energy balance, especially during stress conditions.

3. **Protein Turnover:** Genes LMB90_RS00595 and LMB90_RS02390 are related to posttranslational modifications and protein turnover. These genes may play a role in maintaining protein quality and stability under stress conditions.

4. **Other Functions:** Some genes in this iModulon have functions related to amino acid transport and conversion, transcription, and replication, recombination, and repair.

The presence of CcpA as a regulator suggests a potential connection to carbon catabolite repression (CCR) mechanisms, which allow the cell to prioritize carbon source utilization based on environmental conditions.

Overall, 'uncharacterized iModulon 20' appears to have a diverse role in responding to stress, maintaining energy production, and regulating various cellular processes. It likely helps the cell adapt to changing conditions, but further experimental studies would be needed to confirm its exact biological function and regulatory mechanisms.

Uncharacterized iModulon 21

Jonathan Josephs-Spaulding:

Given the following information, I would like to characterize 'uncharacterized iModulon 21'. Please suggest a name to describe this iModulon and a potential biological function. However, if you cannot make a decision due to limited information, please state that this iModulon is 'Uncharacterized':
,gene_weight,start,end,strand,gene_name,COG,accession,regulator
LMB90_RS00295,0.0588062366445857,8119,8490,ftsL,Op241,"Cell Cycle Control, Cell Division, Chromosome Partitioning",NZ_JAJGVJ010000026.1,
LMB90_RS00300,0.0547535279910181,8490,10649,LMB90_RS00300,Op249,Cell Wall/Membrane/Envelope Biogenesis,NZ_JAJGVJ010000026.1,
LMB90_RS00395,0.0531688675462729,27954,28610,LMB90_RS00395,Op705,Carbohydrate Transport & Metabolism,NZ_JAJGVJ010000026.1,
LMB90_RS00400,0.055993165433588,28623,29306,LMB90_RS00400,Op723,Function Unknown,NZ_JAJGVJ010000026.1,

LMB90_RS00450,0.0782222996210861,3341,4081,-,LMB90_RS00450,Op115,Lipid Transport & Metabolism,NZ_JAJGVJ010000030.1,
LMB90_RS00485,-0.0592573195552433,9381,9782,-,psiE,Op277,Function Unknown,NZ_JAJGVJ010000030.1,
LMB90_RS00490,0.0608605453864386,9914,10684,tpiA,Op295,Carbohydrate Transport & Metabolism,NZ_JAJGVJ010000030.1,
LMB90_RS00690,0.0503513555528684,51098,52057,-,LMB90_RS00690,Op1142,"Replication, Recombination, & Repair",NZ_JAJGVJ010000030.1,
LMB90_RS00880,0.0553985315496027,91308,91877,LMB90_RS00880,Op1679,Function Unknown,NZ_JAJGVJ010000030.1,
LMB90_RS00885,0.0529480303321393,91880,92776,htpX,Op1684,"Posttranslational Modifications, Protein Turnover, Chaperones",NZ_JAJGVJ010000030.1,
LMB90_RS01550,0.0891651506323933,28512,29033,LMB90_RS01550,Op721,Transcription,NZ_JAJGVJ010000044.1,
LMB90_RS01680,0.0569870060560836,8048,8299,LMB90_RS01680,Op237,Function Unknown,NZ_JAJGVJ010000045.1,
LMB90_RS01855,0.0503241019168336,45121,45528,-,LMB90_RS01855,Op1043,Function Unknown,NZ_JAJGVJ010000045.1,
LMB90_RS01920,0.1247187924076903,58289,59239,mvk,Op1251,Lipid Transport & Metabolism,NZ_JAJGVJ010000045.1,
LMB90_RS01925,0.0822794276075987,59239,60210,mvaD,Op1263,Lipid Transport & Metabolism,NZ_JAJGVJ010000045.1,
LMB90_RS01930,0.0670462059196052,60238,61365,LMB90_RS01930,Op1276,Lipid Transport & Metabolism,NZ_JAJGVJ010000045.1,
LMB90_RS01935,0.052151682564467,61381,62427,fni,Op1294,Energy Production & Conversion,NZ_JAJGVJ010000045.1,
LMB90_RS02115,0.0973926991281471,98680,99624,-,rihA,Op1743,Nucleotide Transport & Metabolism,NZ_JAJGVJ010000045.1,
LMB90_RS02320,0.0609588282954381,22557,22775,LMB90_RS02320,Op600,No COG Annotation,NZ_JAJGVJ010000046.1,
LMB90_RS02470,0.0872927146679637,65529,66149,LMB90_RS02470,Op1359,Signal Transduction Mechanisms,NZ_JAJGVJ010000046.1,
LMB90_RS02475,0.0814535943655787,66151,66816,LMB90_RS02475,Op1368,Transcription,NZ_JAJGVJ010000046.1,
LMB90_RS02480,0.07684317347245,66828,68075,LMB90_RS02480,Op1376,Signal Transduction Mechanisms,NZ_JAJGVJ010000046.1,
LMB90_RS02485,0.0724548333172808,68078,69259,LMB90_RS02485,Op1397,Inorganic Ion Transport & Metabolism,NZ_JAJGVJ010000046.1,
LMB90_RS02530,0.1127908793668224,79327,80112,LMB90_RS02530,Op1554,No COG Annotation,NZ_JAJGVJ010000046.1,
LMB90_RS02535,0.0767779237870339,80285,80821,pyrR,Op1564,Nucleotide Transport & Metabolism,NZ_JAJGVJ010000046.1,
LMB90_RS02640,0.0576905594946141,21642,22676,-,LMB90_RS02640,Op577,Transcription,NZ_JAJGVJ010000047.1,
LMB90_RS02670,0.0499719746931976,2680,3897,-,LMB90_RS02670,Op95,Cell Wall/Membrane/Envelope Biogenesis,NZ_JAJGVJ010000048.1,
LMB90_RS02700,0.0729893103991291,9725,10450,LMB90_RS02700,Op285,Carbohydrate Transport & Metabolism,NZ_JAJGVJ010000048.1,
LMB90_RS03455,0.0500679577147574,39653,39832,-,LMB90_RS03455,Op945,No COG Annotation,NZ_JAJGVJ010000051.1,
LMB90_RS03800,0.0642437261802959,112673,112996,-,LMB90_RS03800,Op1834,No COG Annotation,NZ_JAJGVJ010000051.1,
LMB90_RS03815,0.1266735036701146,113938,114321,-,LMB90_RS03815,Op1847,Function Unknown,NZ_JAJGVJ010000051.1,
LMB90_RS03820,0.1227819578595783,114339,114530,-,LMB90_RS03820,Op1849,No COG Annotation,NZ_JAJGVJ010000051.1,
LMB90_RS03825,0.1129584108606803,114545,115090,-,amaP,Op1850,No COG Annotation,NZ_JAJGVJ010000051.1,
LMB90_RS04205,0.0646145129590393,3894,4442,raiA,Op130,"Translation, Ribosomal Structure, & Biogenesis",NZ_JAJGVJ010000055.1,
LMB90_RS04440,-0.0512080642414063,55058,55453,rbsD,Op1199,Carbohydrate Transport & Metabolism,NZ_JAJGVJ010000055.1,"CcpA,CcpA"
LMB90_RS04465,0.0556046102401141,60309,62129,glmS,Op1278,Cell Wall/Membrane/Envelope Biogenesis,NZ_JAJGVJ010000055.1,
LMB90_RS04760,0.051549123905427,128986,130101,LMB90_RS04760,Op1910,Function Unknown,NZ_JAJGVJ010000055.1,
LMB90_RS04935,0.0694421402083192,22715,24601,LMB90_RS04935,Op605,Carbohydrate Transport & Metabolism,NZ_JAJGVJ010000056.1,"CcpA,GalR"
LMB90_RS04940,0.067532170681262,24585,25544,LMB90_RS04940,Op647,Carbohydrate Transport & Metabolism,NZ_JAJGVJ010000056.1,"CcpA,GalR"
LMB90_RS05090,0.0912891486420113,51231,51602,-,mscL,Op1144,Cell Wall/Membrane/Envelope Biogenesis,NZ_JAJGVJ010000056.1,
LMB90_RS05220,0.0499024575838165,22582,23088,-,LMB90_RS05220,Op601,Function Unknown,NZ_JAJGVJ010000057.1,
LMB90_RS05225,0.0604107243746067,23153,23929,-,LMB90_RS05225,Op617,"Translation, Ribosomal Structure, & Biogenesis",NZ_JAJGVJ010000057.1,
LMB90_RS05285,0.0716327381443417,3366,3920,LMB90_RS05285,Op116,Transcription,NZ_JAJGVJ010000058.1,
LMB90_RS05370,0.0703579405942092,21918,22484,-,LMB90_RS05370,Op584,Defense Mechanisms,NZ_JAJGVJ010000058.1,
LMB90_RS05575,0.0624383391803935,60382,61119,LMB90_RS05575,Op1280,Defense Mechanisms,NZ_JAJGVJ010000058.1,
LMB90_RS05580,0.0648366789768912,61112,62314,LMB90_RS05580,Op1290,"Intracellular Trafficking, Secretion, & Vesicular Transport",NZ_JAJGVJ010000058.1,

LMB90_RS05585,0.061382047404104,62327,62968,trmB,Op1309,"Translation, Ribosomal Structure, &
Biogenesis",NZ_JAJGVJ010000058.1,
LMB90_RS05600,-0.0529574093303164,67754,67960,LMB90_RS05600,Op1393,No COG Annotation,NZ_JAJGVJ010000058.1,
LMB90_RS05640,0.0833082124018616,74790,75503,LMB90_RS05640,Op1495,Function Unknown,NZ_JAJGVJ010000058.1,
LMB90_RS05785,0.0934857776802199,569,1216,LMB90_RS05785,Op42,Function Unknown,NZ_JAJGVJ010000059.1,
LMB90_RS05910,0.0499650686379674,19242,19796,LMB90_RS05910,Op530,Function Unknown,NZ_JAJGVJ010000059.1,
LMB90_RS06245,0.0516509537799509,99552,99776,-,LMB90_RS06245,Op1751,Function Unknown,NZ_JAJGVJ010000059.1,
LMB90_RS06255,0.0507572993254745,100043,101035,-,LMB90_RS06255,Op1756,"Cell Cycle Control, Cell Division,
Chromosome Partitioning",NZ_JAJGVJ010000059.1,
LMB90_RS06260,0.0658742389089731,101142,102458,-,murA,Op1764,Cell Wall/Membrane/Envelope
Biogenesis,NZ_JAJGVJ010000059.1,
LMB90_RS06265,0.0716723269454074,102477,102740,-,LMB90_RS06265,Op1775,Function Unknown,NZ_JAJGVJ010000059.1,
LMB90_RS07250,0.1254106392212971,69594,70061,-,LMB90_RS07250,Op1417,Function Unknown,NZ_JAJGVJ010000062.1,
LMB90_RS07370,0.0976907734622458,96030,96680,-,LMB90_RS07370,Op1719,Function Unknown,NZ_JAJGVJ010000062.1,
LMB90_RS07395,0.0652199903910044,103215,105311,LMB90_RS07395,Op1779,Energy Production &
Conversion,NZ_JAJGVJ010000062.1,
LMB90_RS07475,0.0578667503946942,116,520,-,LMB90_RS07475,Op26,No COG Annotation,NZ_JAJGVJ010000063.1,
LMB90_RS07520,0.0761712659181009,15150,16160,-,LMB90_RS07520,Op431,Nucleotide Transport &
Metabolism,NZ_JAJGVJ010000063.1,CcpA
LMB90_RS07580,-0.0742310121188032,60104,61309,LMB90_RS07580,Op1274,Amino Acid Transport &
Conversion,NZ_JAJGVJ010000063.1,DeoR
LMB90_RS07585,-0.0829549822612212,61390,62298,rihC,Op1296,Amino Acid Transport & Conversion,NZ_JAJGVJ010000063.1,
LMB90_RS07660,0.0807273329935734,75785,76474,LMB90_RS07660,Op1508,Transcription,NZ_JAJGVJ010000063.1,
LMB90_RS07665,0.073441314780578,76506,77651,LMB90_RS07665,Op1517,Nucleotide Transport &
Metabolism,NZ_JAJGVJ010000063.1,
LMB90_RS07740,0.0584396663136066,4947,5834,LMB90_RS07740,Op154,"Posttranslational Modifications, Protein Turnover,
Chaperones",NZ_JAJGVJ010000064.1,
LMB90_RS07745,0.061406169098291,5824,6852,LMB90_RS07745,Op180,Function Unknown,NZ_JAJGVJ010000064.1,
LMB90_RS08140,0.0715030894674962,2786,4192,-,LMB90_RS08140,Op97,No COG Annotation,NZ_JAJGVJ010000065.1,
LMB90_RS08445,0.0916043589307106,4616,5617,LMB90_RS08445,Op148,Transcription,NZ_JAJGVJ010000066.1,
LMB90_RS08580,0.1392748712691725,31507,31740,LMB90_RS08580,Op782,Carbohydrate Transport &
Metabolism,NZ_JAJGVJ010000066.1,
LMB90_RS08655,0.0957765231119902,49038,51020,LMB90_RS08655,Op1109,Defense Mechanisms,NZ_JAJGVJ010000066.1,
LMB90_RS08665,0.078884176834811,51470,51787,-,LMB90_RS08665,Op1150,Defense Mechanisms,NZ_JAJGVJ010000066.1,
LMB90_RS08670,-0.0770657995522777,51951,53666,-,LMB90_RS08670,Op1158,No COG Annotation,NZ_JAJGVJ010000066.1,
LMB90_RS08685,0.0603544311598812,55579,56199,LMB90_RS08685,Op1209,Transcription,NZ_JAJGVJ010000066.1,
LMB90_RS08705,0.0687762768898673,59713,60351,-,LMB90_RS08705,Op1270,"Translation, Ribosomal Structure, &
Biogenesis",NZ_JAJGVJ010000066.1,
LMB90_RS08735,0.0821719125362236,66133,67254,-,LMB90_RS08735,Op1366,Cell Wall/Membrane/Envelope
Biogenesis,NZ_JAJGVJ010000066.1,Rex
LMB90_RS08785,0.0772529990883788,75431,77560,-,LMB90_RS08785,Op1502,Amino Acid Transport &
Conversion,NZ_JAJGVJ010000066.1,
LMB90_RS08790,0.1185765948324275,77686,77949,-,LMB90_RS08790,Op1529,Nucleotide Transport &
Metabolism,NZ_JAJGVJ010000066.1,
LMB90_RS09015,0.0587071192331995,120908,122812,LMB90_RS09015,Op1885,Function Unknown,NZ_JAJGVJ010000066.1,
LMB90_RS09135,0.1035520511145257,145181,145792,-,LMB90_RS09135,Op1942,Carbohydrate Transport &
Metabolism,NZ_JAJGVJ010000066.1,
LMB90_RS09615,0.0735083364362283,37164,37925,-,LMB90_RS09615,Op898,Cell Wall/Membrane/Envelope
Biogenesis,NZ_JAJGVJ010000068.1,
LMB90_RS09620,0.0768114505468727,38003,39775,-,LMB90_RS09620,Op915,Carbohydrate Transport &
Metabolism,NZ_JAJGVJ010000068.1,
LMB90_RS09715,0.0566507289252917,62394,63191,-,LMB90_RS09715,Op1312,Amino Acid Transport &
Conversion,NZ_JAJGVJ010000068.1,
LMB90_RS09755,0.056345578719804,73078,74616,LMB90_RS09755,Op1470,No COG Annotation,NZ_JAJGVJ010000068.1,
LMB90_RS09965,0.0618186826623642,40516,41439,LMB90_RS09965,Op960,"Replication, Recombination, &
Repair",NZ_JAJGVJ010000069.1,
LMB90_RS10085,0.0628273902759891,61192,62082,LMB90_RS10085,Op1291,Amino Acid Transport &
Conversion,NZ_JAJGVJ010000069.1,

ChatGPT:
Based on the provided information, we can characterize 'uncharacterized iModulon 21' as follows:

Name: Isoprenoid Biosynthesis and Adaptive Response iModulon

Potential Biological Function: Regulation of genes involved in isoprenoid biosynthesis and the adaptive response to environmental stress, particularly related to the production of mevalonate and other isoprenoids that are essential for various cellular processes. This iModulon may play a role in responding to changes in environmental conditions, such as stress, and coordinating the biosynthesis of isoprenoid compounds critical for cell biology.

This name and function are consistent with the genes and processes you've listed, including those related to isoprenoid biosynthesis (mvaD, mvk), cell division and wall synthesis (ftsL), stress responses (htpX, mscL), and various metabolic pathways. However, it's important to note that further experimental validation and research would be needed to confirm the precise role and regulatory mechanisms of this iModulon.

Uncharacterized iModulon 25
Jonathan Josephs-Spaulding:
Given the following information, I would like to characterize 'uncharacterized iModulon 25. Please suggest a name to describe this iModulon and a potential biological function. However, if you cannot make a decision due to limited information, please state that this iModulon is 'Uncharacterized': ,gene_weight,start,end,strand,gene_name,COG,accession,regulator LMB90_RS05435,0.120214114165483,36940,37572,-,LMB90_RS05435,Op894,Nucleotide Transport & Metabolism,NZ_JAJGVJ010000058.1,NrdR LMB90_RS07565,0.130606431261916,55286,56749,LMB90_RS07565,Op1205,Cell Wall/Membrane/Envelope Biogenesis,NZ_JAJGVJ010000063.1, LMB90_RS07570,0.1394583311686482,56751,57494,LMB90_RS07570,Op1229,"Translation, Ribosomal Structure, & Biogenesis",NZ_JAJGVJ010000063.1, LMB90_RS09830,0.1062205082966169,15135,15299,LMB90_RS09830,Op429,"Translation, Ribosomal Structure, & Biogenesis",NZ_JAJGVJ010000069.1, LMB90_RS10095,0.1035452719997846,62383,62598,-,LMB90_RS10095,Op1310,Function Unknown,NZ_JAJGVJ010000069.1,
ChatGPT:
Based on the limited information provided, it's not possible to confidently characterize 'iModulon 25.' Therefore, this iModulon remains 'Uncharacterized-2' in terms of both its name and potential biological function.

Uncharacterized iModulon 26
Jonathan Josephs-Spaulding:
Given the following information, I would like to characterize 'uncharacterized iModulon 26. Please suggest a name to describe this iModulon and a potential biological function. However, if you cannot make a decision due to limited information, please state that this iModulon is 'Uncharacterized': ,gene_weight,start,end,strand,gene_name,COG,accession,regulator LMB90_RS01145,0.1578338388396872,1,246,-,LMB90_RS01145,Op16,No COG Annotation,NZ_JAJGVJ010000038.1, LMB90_RS01440,0.1417127698063508,160,671,LMB90_RS01440,Op34,"Replication, Recombination, & Repair",NZ_JAJGVJ010000043.1, LMB90_RS02330,0.1655801121219139,23604,24776,LMB90_RS02330,Op627,Energy Production & Conversion,NZ_JAJGVJ010000046.1,"CcpA,Rex" LMB90_RS09170,0.1697295870838096,152398,153030,-,LMB90_RS09170,Op1949,Carbohydrate Transport & Metabolism,NZ_JAJGVJ010000066.1,

ChatGPT:
Based on the limited information provided, it's difficult to definitively characterize 'iModulon 26.' Based on the limited information provided, it's not possible to confidently characterize 'iModulon 26.' Therefore, this iModulon remains 'Uncharacterized-3' in terms of both its name and potential biological function. However, we can make some observations:

1. **LMB90_RS01145:** This gene has no COG annotation, which means its function is currently unknown.

2. **LMB90_RS01440:** This gene is associated with "Replication, Recombination, & Repair," suggesting a potential role in DNA-related processes.

3. **LMB90_RS02330:** This gene is associated with "Energy Production & Conversion" and is regulated by both CcpA and Rex, which implies a role in energy metabolism.

4. **LMB90_RS09170:** This gene is associated with "Carbohydrate Transport & Metabolism," indicating involvement in carbohydrate metabolism.

Given the diverse functions of these genes, it's challenging to assign a specific function to 'iModulon 26' without more information. Therefore, this iModulon remains 'Uncharacterized' in terms of both its name and potential biological function.

Uncharacterized iModulon 27
Jonathan Josephs-Spaulding:
Given the following information, I would like to characterize 'uncharacterized iModulon 27. Please suggest a name to describe this iModulon and a potential biological function. However, if you cannot make a decision due to limited information, please state that this iModulon is 'Uncharacterized':
,gene_weight,start,end,strand,gene_name,COG,accession,regulator
LMB90_RS02710,0.1486115867665163,11194,12060,-,LMB90_RS02710,Op332,Defense Mechanisms,NZ_JAJGVJ010000048.1,
LMB90_RS02715,0.2155028058552994,12038,12421,-,LMB90_RS02715,Op351,Transcription,NZ_JAJGVJ010000048.1,
LMB90_RS02780,-0.1204989121615769,6820,8214,LMB90_RS02780,Op201,Energy Production & Conversion,NZ_JAJGVJ010000050.1,

ChatGPT:
Based on the limited information provided, it's difficult to definitively characterize 'iModulon 27.' Based on the limited information provided, it's not possible to confidently characterize 'iModulon 27.' Therefore, this iModulon remains 'Uncharacterized-4' in terms of both its name and potential biological function. However, we can make some observations:

1. **LMB90_RS02710:** This gene is associated with "Defense Mechanisms," suggesting a potential role in the defense response of the organism.

2. **LMB90_RS02715:** This gene is associated with "Transcription," indicating a role in regulating gene expression.

3. **LMB90_RS02780:** This gene is associated with "Energy Production & Conversion," implying involvement in energy metabolism.

Given the diverse functions of these genes, it's difficult to assign a specific function to 'iModulon 27' without more information. Therefore, this iModulon remains 'Uncharacterized 4' in terms of both its name and potential biological function.

Uncharacterized iModulon 28
Jonathan Josephs-Spaulding:
Given the following information, I would like to characterize 'uncharacterized iModulon 28. Please suggest a name to describe this iModulon and a potential biological function. However, if you cannot make a decision due to limited information, please state that this iModulon is 'Uncharacterized':
,gene_weight,start,end,strand,gene_name,COG,accession,regulator
LMB90_RS01535,0.1071448053893864,25502,26662,LMB90_RS01535,Op663,Amino Acid Transport & Conversion,NZ_JAJGVJ010000044.1,
LMB90_RS01615,0.1502369905759518,325,678,-,LMB90_RS01615,Op35,No COG Annotation,NZ_JAJGVJ010000045.1,
LMB90_RS01960,0.1084531304620111,67469,68443,-,LMB90_RS01960,Op1386,Energy Production & Conversion,NZ_JAJGVJ010000045.1,
LMB90_RS01965,0.1119058090926666,68471,69211,-,LMB90_RS01965,Op1402,Cell Wall/Membrane/Envelope Biogenesis,NZ_JAJGVJ010000045.1,CcpA
LMB90_RS01970,0.1006351315925609,69204,69623,-,LMB90_RS01970,Op1411,Function Unknown,NZ_JAJGVJ010000045.1,CcpA
LMB90_RS02055,0.1007003928134418,86042,86200,LMB90_RS02055,Op1626,No COG Annotation,NZ_JAJGVJ010000045.1,
LMB90_RS02185,0.1817245471014277,112910,113401,LMB90_RS02185,Op1836,Function Unknown,NZ_JAJGVJ010000045.1,
LMB90_RS05360,0.0975968229472171,21045,21569,-,LMB90_RS05360,Op565,Function Unknown,NZ_JAJGVJ010000058.1,
LMB90_RS07515,0.1034616294252865,13616,14989,-,LMB90_RS07515,Op397,Nucleotide Transport & Metabolism,NZ_JAJGVJ010000063.1,
LMB90_RS08335,0.0931121627644041,45502,45759,LMB90_RS08335,Op1053,Coenzyme Transport & Metabolism,NZ_JAJGVJ010000065.1,
LMB90_RS08400,0.1281670154245571,60683,61519,LMB90_RS08400,Op1285,"Cell Cycle Control, Cell Division, Chromosome Partitioning",NZ_JAJGVJ010000065.1,
LMB90_RS08960,0.1275082168817138,112911,113195,-,LMB90_RS08960,Op1837,No COG Annotation,NZ_JAJGVJ010000066.1,

LMB90_RS09060,-0.0938295439346036,131721,132476,-,LMB90_RS09060,Op1915,Amino Acid Transport & Conversion,NZ_JAJGVJ010000066.1,
LMB90_RS10090,0.0989209452453911,62134,62361,-,LMB90_RS10090,Op1305,Amino Acid Transport & Conversion,NZ_JAJGVJ010000069.1,

ChatGPT:
Based on the limited information provided, it's difficult to definitively characterize 'iModulon 28.' Based on the limited information provided, it's not possible to confidently characterize 'iModulon 28.' Therefore, this iModulon remains 'Uncharacterized-5' in terms of both its name and potential biological function. However, we can make some observations:

1. **LMB90_RS01535:** This gene is associated with "Amino Acid Transport & Conversion," suggesting a potential role in amino acid metabolism and transport.

2. **LMB90_RS01615:** This gene has "No COG Annotation," indicating that its function is currently unknown.

3. **LMB90_RS01960:** This gene is associated with "Energy Production & Conversion," implying involvement in energy metabolism.

4. **LMB90_RS01965:** This gene is associated with "Cell Wall/Membrane/Envelope Biogenesis," suggesting a potential role in cell envelope formation.

5. **LMB90_RS01970:** This gene has an "Unknown Function" and is located in the same operon as LMB90_RS01965.

6. **LMB90_RS02055:** This gene also has "No COG Annotation," indicating an unknown function.

7. **LMB90_RS02185:** This gene is categorized as "Function Unknown."

8. **LMB90_RS05360:** This gene is categorized as "Function Unknown" and is regulated by NrdR.

9. **LMB90_RS07515:** This gene is associated with "Nucleotide Transport & Metabolism," suggesting a role in nucleotide metabolism.

10. **LMB90_RS08335:** This gene is associated with "Coenzyme Transport & Metabolism," indicating a potential role in coenzyme transport and metabolism.

11. **LMB90_RS08400:** This gene is associated with "Cell Cycle Control, Cell Division, Chromosome Partitioning," suggesting involvement in cell cycle regulation.

12. **LMB90_RS08960:** This gene has "No COG Annotation" and is regulated by NrdR.

13. **LMB90_RS09060:** This gene is associated with "Amino Acid Transport & Conversion," indicating a role in amino acid metabolism and transport.

14. **LMB90_RS10090:** This gene is associated with "Amino Acid Transport & Conversion," suggesting a potential role in amino acid metabolism and transport.

Given the diversity of functions and the presence of genes with unknown functions in this iModulon, it's challenging to assign a specific name or biological function to 'iModulon 28.' Therefore, this iModulon remains 'Uncharacterized' in terms of both its name and potential biological function.

Uncharacterized iModulon 29
Jonathan Josephs-Spaulding:
Given the following information, I would like to characterize 'uncharacterized iModulon 29. Please suggest a name to describe this iModulon and a potential biological function. However, if you cannot make a decision due to limited information, please state that this iModulon is 'Uncharacterized':
,gene_weight,start,end,strand,gene_name,COG,accession,regulator
LMB90_RS01580,0.1128591696352773,34673,35149,LMB90_RS01580,Op850,Coenzyme Transport & Metabolism,NZ_JAJGVJ010000044.1,T-box(Met)
LMB90_RS01585,0.105899784288173,35265,36128,LMB90_RS01585,Op861,Inorganic Ion Transport & Metabolism,NZ_JAJGVJ010000044.1,T-box(Met)
LMB90_RS04265,0.0824999929801062,16111,17043,trxB,Op455,Energy Production & Conversion,NZ_JAJGVJ010000055.1,

LMB90_RS04960,0.1835791420038246,29407,30549,LMB90_RS04960,Op739,Amino Acid Transport & Conversion,NZ_JAJGVJ010000056.1,
LMB90_RS04965,0.193813509552857,30576,31277,LMB90_RS04965,Op761,Amino Acid Transport & Conversion,NZ_JAJGVJ010000056.1,
LMB90_RS04970,0.1703771646154654,31283,32032,LMB90_RS04970,Op778,Amino Acid Transport & Conversion,NZ_JAJGVJ010000056.1,
LMB90_RS04975,0.1751279768401976,32048,32839,LMB90_RS04975,Op798,Amino Acid Transport & Conversion,NZ_JAJGVJ010000056.1,
LMB90_RS06025,0.0967900779108836,46872,47723,-,LMB90_RS06025,Op1075,Amino Acid Transport & Conversion,NZ_JAJGVJ010000059.1,
LMB90_RS06030,0.1163184381214753,47741,48391,-,LMB90_RS06030,Op1089,Amino Acid Transport & Conversion,NZ_JAJGVJ010000059.1,
LMB90_RS06035,0.1118728388608122,48406,49053,-,LMB90_RS06035,Op1100,Inorganic Ion Transport & Metabolism,NZ_JAJGVJ010000059.1,
LMB90_RS06780,0.0792231557694122,97668,98240,grpE,Op1734,Transcription,NZ_JAJGVJ010000060.1,"HrcA,CtsR"
LMB90_RS08560,0.0965358828988263,28967,29569,-,LMB90_RS08560,Op729,No COG Annotation,NZ_JAJGVJ010000066.1,
LMB90_RS09410,0.0797793176950016,46469,47239,-,LMB90_RS09410,Op1065,"Translation, Ribosomal Structure, & Biogenesis",NZ_JAJGVJ010000067.1,
LMB90_RS09495,-0.0828951899532132,16344,16739,LMB90_RS09495,Op463,Transcription,NZ_JAJGVJ010000068.1,
LMB90_RS09665,0.146490701824768,49555,50469,LMB90_RS09665,Op1116,Inorganic Ion Transport & Metabolism,NZ_JAJGVJ010000068.1,

ChatGPT:
Name: Stress Response and Proteostasis iModulon

Biological Function: The 'Stress Response and Proteostasis' iModulon likely plays a role in responding to cellular stress and maintaining proteostasis (protein homeostasis). This iModulon comprises genes associated with various functions:

1. Coenzyme Transport & Metabolism: Genes related to coenzyme transport and metabolism may be involved in providing essential coenzymes necessary for various cellular processes, including energy production and biosynthesis.

2. Inorganic Ion Transport & Metabolism: The presence of genes associated with inorganic ion transport and metabolism suggests a potential role in regulating the intracellular concentration of ions, which is crucial for cellular homeostasis.

3. Amino Acid Transport & Conversion: Several genes related to amino acid transport and conversion are present, indicating a potential role in regulating amino acid metabolism, which is essential for protein synthesis and cellular growth.

4. Transcription: Genes involved in transcription regulation are part of this iModulon. These genes may play a role in responding to stress conditions by controlling the expression of stress-related genes.

5. Translation, Ribosomal Structure, & Biogenesis: The presence of genes associated with translation and ribosomal structure suggests a potential role in regulating protein synthesis, which is essential for maintaining cellular functions, especially under stress conditions.

6. No COG Annotation: Some genes within this iModulon do not have a known Cluster of Orthologous Groups (COG) annotation, indicating that their functions remain uncharacterized.

The regulation of this iModulon by "HrcA" and "CtsR," both of which are associated with heat shock response genes, further supports its potential involvement in responding to various stress conditions, including thermal stress. However, the exact mechanisms and regulatory pathways involved in the stress response and proteostasis remain to be elucidated through further experimental studies.

Uncharacterized iModulon 30
Jonathan Josephs-Spaulding:
Given the following information, I would like to characterize 'uncharacterized iModulon 30. Please suggest a name to describe this iModulon and a potential biological function. However, if you cannot make a decision due to limited information, please state that this iModulon is 'Uncharacterized': ,gene_weight,start,end,strand,gene_name,COG,accession,regulator
LMB90_RS00480,-0.0974137227031594,8827,9369,-,LMB90_RS00480,Op259,Function Unknown,NZ_JAJGVJ010000030.1,
LMB90_RS01335,0.0951076959725306,28084,28356,LMB90_RS01335,Op707,No COG Annotation,NZ_JAJGVJ010000039.1,

LMB90_RS01510,0.1204892388716003,17887,18459,-,LMB90_RS01510,Op498,Function Unknown,NZ_JAJGVJ010000044.1,
LMB90_RS01630,0.2802624131168066,2734,3006,-,LMB90_RS01630,Op96,Transcription,NZ_JAJGVJ010000045.1,
LMB90_RS01635,0.1180597236401281,3006,3371,-,LMB90_RS01635,Op104,Function Unknown,NZ_JAJGVJ010000045.1,
LMB90_RS02055,0.0833648241556252,86042,86200,LMB90_RS02055,Op1626,No COG Annotation,NZ_JAJGVJ010000045.1,
LMB90_RS02240,0.0935482937101263,6793,7119,LMB90_RS02240,Op198,No COG Annotation,NZ_JAJGVJ010000046.1,
LMB90_RS02540,-0.0957630569295194,80916,81302,-,LMB90_RS02540,Op1574,No COG Annotation,NZ_JAJGVJ010000046.1,
LMB90_RS02545,0.1380367218506713,82017,82658,LMB90_RS02545,Op1583,Transcription,NZ_JAJGVJ010000046.1,
LMB90_RS04085,0.0849392165128155,2574,3104,-,LMB90_RS04085,Op90,Function Unknown,NZ_JAJGVJ010000053.1,
LMB90_RS05405,0.0982797566696674,30787,31050,LMB90_RS05405,Op765,No COG Annotation,NZ_JAJGVJ010000058.1,
LMB90_RS05475,0.1448956211658969,42363,42758,LMB90_RS05475,Op993,No COG Annotation,NZ_JAJGVJ010000058.1,
LMB90_RS05825,0.1644527831709354,6766,7056,-,LMB90_RS05825,Op196,No COG Annotation,NZ_JAJGVJ010000059.1,
LMB90_RS07460,0.110081362276972,119649,120494,-,LMB90_RS07460,Op1880,Function Unknown,NZ_JAJGVJ010000062.1,
LMB90_RS08325,0.1055677115056486,43095,44027,mmuM,Op1010,Function Unknown,NZ_JAJGVJ010000065.1,T-box(Met)
LMB90_RS09410,0.0818472295414384,46469,47239,-,LMB90_RS09410,Op1065,"Translation, Ribosomal Structure, & Biogenesis",NZ_JAJGVJ010000067.1,

ChatGPT:
Based on the limited information provided, it is challenging to definitively characterize 'uncharacterized iModulon 30' with a specific name and biological function. Therefore, I will classify this iModulon as 'Uncharacterized-6' due to the lack of sufficient data to provide a specific name and biological function.

Uncharacterized iModulon 31
Jonathan Josephs-Spaulding:
Given the following information, I would like to characterize 'uncharacterized iModulon 31. Please suggest a name to describe this iModulon and a potential biological function. However, if you cannot make a decision due to limited information, please state that this iModulon is 'Uncharacterized':
,gene_weight,start,end,strand,gene_name,COG,accession,regulator
LMB90_RS01355,0.1212440723109835,31548,31793,-,LMB90_RS01355,Op783,Coenzyme Transport & Metabolism,NZ_JAJGVJ010000039.1,
LMB90_RS01645,-0.0989848104824319,3924,4286,LMB90_RS01645,Op133,Function Unknown,NZ_JAJGVJ010000045.1,
LMB90_RS01675,0.1432598591948374,7488,7931,-,LMB90_RS01675,Op223,Function Unknown,NZ_JAJGVJ010000045.1,
LMB90_RS01730,-0.1269342744434319,21218,21595,-,LMB90_RS01730,Op569,"Cell Cycle Control, Cell Division, Chromosome Partitioning",NZ_JAJGVJ010000045.1,
LMB90_RS02175,0.1036718690375988,111534,111773,LMB90_RS02175,Op1826,Transcription,NZ_JAJGVJ010000045.1,
LMB90_RS04200,0.0933244992945834,3340,3762,LMB90_RS04200,Op114,Function Unknown,NZ_JAJGVJ010000055.1,
LMB90_RS05790,0.1272723148275116,1184,1837,-,LMB90_RS05790,Op52,Function Unknown,NZ_JAJGVJ010000059.1,
LMB90_RS07105,0.0984959727588707,38692,39402,-,LMB90_RS07105,Op932,Transcription,NZ_JAJGVJ010000062.1,
LMB90_RS08170,0.1359568046046113,9030,9605,-,LMB90_RS08170,Op268,No COG Annotation,NZ_JAJGVJ010000065.1,
LMB90_RS09165,0.0960213084777993,151999,152265,-,LMB90_RS09165,Op1948,Function Unknown,NZ_JAJGVJ010000066.1,
LMB90_RS09335,0.1113563550256601,36170,36493,LMB90_RS09335,Op880,Transcription,NZ_JAJGVJ010000067.1,
LMB90_RS09495,-0.1868930090183154,16344,16739,LMB90_RS09495,Op463,Transcription,NZ_JAJGVJ010000068.1,
LMB90_RS10095,0.3721409033685992,62383,62598,-,LMB90_RS10095,Op1310,Function Unknown,NZ_JAJGVJ010000069.1,

ChatGPT:
Based on the limited information provided, it is challenging to definitively characterize 'uncharacterized iModulon 31' with a specific name and biological function. Therefore, I will classify this iModulon as 'Uncharacterized-7' due to the lack of sufficient data to provide a specific name and biological function.
Uncharacterized iModulon 32
Jonathan Josephs-Spaulding:
Given the following information, I would like to characterize 'uncharacterized iModulon 32. Please suggest a name to describe this iModulon and a potential biological function. However, if you cannot make a decision due to limited information, please state that this iModulon is 'Uncharacterized':
,gene_weight,start,end,strand,gene_name,COG,accession,regulator
LMB90_RS01560,0.0816934784248637,30771,31346,LMB90_RS01560,Op763,Function Unknown,NZ_JAJGVJ010000044.1,
LMB90_RS01630,0.30251255474471,2734,3006,-,LMB90_RS01630,Op96,Transcription,NZ_JAJGVJ010000045.1,
LMB90_RS01645,0.2819886302211395,3924,4286,LMB90_RS01645,Op133,Function Unknown,NZ_JAJGVJ010000045.1,
LMB90_RS01670,-0.0926609470535682,6840,7478,-,LMB90_RS01670,Op202,Function Unknown,NZ_JAJGVJ010000045.1,
LMB90_RS02765,-0.1593308687368612,2370,3251,-,LMB90_RS02765,Op84,Carbohydrate Transport & Metabolism,NZ_JAJGVJ010000050.1,MleR
LMB90_RS03015,0.0834346534902788,56002,56676,-,LMB90_RS03015,Op1217,Cell Motility,NZ_JAJGVJ010000050.1,

LMB90_RS04525,0.0946363051366742,72987,73196,LMB90_RS04525,Op1469,No COG Annotation,NZ_JAJGVJ010000055.1,
LMB90_RS05775,-0.277760652980305,98392,98667,-,LMB90_RS05775,Op1740,Energy Production & Conversion,NZ_JAJGVJ010000058.1,
LMB90_RS05835,0.1538398277906155,7474,7785,-,LMB90_RS05835,Op221,"Intracellular Trafficking, Secretion, & Vesicular Transport",NZ_JAJGVJ010000059.1,
LMB90_RS06490,0.1371073755504224,44492,44629,-,LMB90_RS06490,Op1033,No COG Annotation,NZ_JAJGVJ010000060.1,
LMB90_RS08190,0.0942957588758274,12466,13341,LMB90_RS08190,Op362,Transcription,NZ_JAJGVJ010000065.1,
LMB90_RS09165,-0.0976336262755775,151999,152265,-,LMB90_RS09165,Op1948,Function Unknown,NZ_JAJGVJ010000066.1,
LMB90_RS09410,-0.0875544025418511,46469,47239,-,LMB90_RS09410,Op1065,"Translation, Ribosomal Structure, & Biogenesis",NZ_JAJGVJ010000067.1,
LMB90_RS09945,-0.0919934524848903,37682,37867,-,LMB90_RS09945,Op908,Energy Production & Conversion,NZ_JAJGVJ010000069.1,

ChatGPT:
Based on the limited information provided, it is challenging to definitively characterize 'uncharacterized iModulon 32' with a specific name and biological function. Therefore, I will classify this iModulon as 'Uncharacterized-8' due to the lack of sufficient data to provide a specific name and biological function.

Uncharacterized iModulon 33

Jonathan Josephs-Spaulding:
Given the following information, I would like to characterize 'uncharacterized iModulon 33. Please suggest a name to describe this iModulon and a potential biological function. However, if you cannot make a decision due to limited information, please state that this iModulon is 'Uncharacterized':
,gene_weight,start,end,strand,gene_name,COG,accession,regulator
LMB90_RS01345,0.1482462202903757,29335,30243,LMB90_RS01345,Op737,Coenzyme Transport & Metabolism,NZ_JAJGVJ010000039.1,
LMB90_RS03815,0.1412805749796894,113938,114321,-,LMB90_RS03815,Op1847,Function Unknown,NZ_JAJGVJ010000051.1,
LMB90_RS03820,0.145083417198049,114339,114530,-,LMB90_RS03820,Op1849,No COG Annotation,NZ_JAJGVJ010000051.1,
LMB90_RS03825,0.1611201922929264,114545,115090,-,amaP,Op1850,No COG Annotation,NZ_JAJGVJ010000051.1,
LMB90_RS04680,0.1000323969635354,112001,112600,-,LMB90_RS04680,Op1830,No COG Annotation,NZ_JAJGVJ010000055.1,
LMB90_RS05820,-0.1023259063435501,6338,6658,-,LMB90_RS05820,Op192,No COG Annotation,NZ_JAJGVJ010000059.1,
LMB90_RS08020,0.100989297271098,56035,56259,-,LMB90_RS08020,Op1218,No COG Annotation,NZ_JAJGVJ010000064.1,
LMB90_RS08580,0.1228926177208206,31507,31740,LMB90_RS08580,Op782,Carbohydrate Transport & Metabolism,NZ_JAJGVJ010000066.1,
LMB90_RS09165,-0.1018059678768408,151999,152265,-,LMB90_RS09165,Op1948,Function Unknown,NZ_JAJGVJ010000066.1,
LMB90_RS09945,0.1519024121734351,37682,37867,-,LMB90_RS09945,Op908,Energy Production & Conversion,NZ_JAJGVJ010000069.1,
LMB90_RS09970,0.1350274200577635,41446,41892,-,LMB90_RS09970,Op978,Energy Production & Conversion,NZ_JAJGVJ010000069.1,
LMB90_RS09975,0.1808242450733174,41889,42323,-,LMB90_RS09975,Op983,Cell Wall/Membrane/Envelope Biogenesis,NZ_JAJGVJ010000069.1,

ChatGPT:
Based on the limited information provided, it is challenging to definitively characterize 'uncharacterized iModulon 33' with a specific name and biological function. Therefore, I will classify this iModulon as 'Uncharacterized-9' due to the lack of sufficient data to provide a specific name and biological function.
