# Supplementary material for: Reconstructing the transcriptional regulatory network of probiotic L. reuteri is enabled by transcriptomics and machine learning
Source: mSystems. 2024 Feb 13;9(3):e01257-23. doi: 10.1128/msystems.01257-23 (PMC10949432; doi:10.1128/msystems.01257-23)
Supplement: Supplemental Discussion — Discussion of the 10 identified functional iModulons. [file msystems.01257-23-s0003.docx]

**Reconstructing the Transcriptional Regulatory Network of Probiotic *L. reuteri* is Enabled by Transcriptomics and Machine Learning**

Jonathan Josephs-Spaulding^1^, Akanksha Rajput^2^, Ying Hefner^2^, Richard Szubin^2^, Archana Balasubramanian^2^, Gaoyuan Li^2^, Daniel C. Zielinski^2^, Leonie Jahn^1^, Morten Sommer^1^, Patrick Phaneuf^1^, Bernhard O. Palsson^1,2*^

^1^ The Novo Nordisk Foundation Center for Biosustainability, Technical University of

Denmark, Copenhagen, Denmark

^2^ Department of Bioengineering, University of California, San Diego, La Jolla, CA, United States

^*^ Correspondence: palsson@ucsd.edu

**Supplementary Discussion**

This study's significant finding is the identification of 11 unique Functional iModulons from 20 Uncharacterized ones in *L. reuteri*. iModulons enriched with gene clusters for specific biological roles that are not yet linked to known transcriptional regulators offer a promising area for future research. Their coordinated response to environmental stimuli underscores their potential to reveal novel regulatory mechanisms and advance microbial strain design and are described:

- The "Stress Response and Homeostasis" iModulon in *L. reuteri* integrates stress adaptation and metabolic regulation with genes like *mscL* for stress response (1), *rfbB* for riboflavin synthesis (2), and *psiE* for RNA modification (3). It also includes genes for signal transduction, cell integrity, and repair, influenced by regulators like PLP-aminotransferase, known for anti-inflammatory effects in liver conditions (4). This gene diversity suggests its role in cellular equilibrium and stress resilience, warranting further exploration of its regulatory roles and potential in therapy. The "Cell Envelope and Transport-Related” iModulon in *L. reuteri* includes genes like *LMB90_RS04490* and *LMB90_RS05310*, which were annotated to play roles in cell wall integrity and envelope biogenesis, and *LMB90_RS08335*, associated with coenzyme transport and metabolism. Additionally, *MalR*-regulated *LMB90_RS02450* highlights its role in carbohydrate metabolism, adapting to environmental nutrients. Further research is needed to understand how these genes affect cell envelope dynamics and metabolic efficiency.'
- The "RP-Operon-1" iModulon in *L. reuteri*, with genes like *rpsL* and *rplC*, is pivotal for ribosome assembly, underscoring a robust commitment to protein synthesis for growth or heightened protein demand (5). In contrast, "RP-Operon-2," featuring *rpmA*, *rpmB*, and *rpmH*, may finely tune this synthesis, adapting to particular environmental or developmental signals (6,7,8). While both iModulons are crucial to protein production, their unique gene sets hint at diverse roles in cellular adaptive mechanisms, with implications for antibiotic interactions (9), meriting deeper functional analysis.
- The "Nitrate Reductase and Cofactor Metabolism” iModulon, with the NAR operon (*narI*, *narJ*, *narH*), and *mobB*, is central to *L. reuteri's* anaerobic respiration and molybdenum cofactor-dependent processes like nitrate assimilation (10, 11, 12). It includes coenzyme metabolism genes and *preA*, potentially involved in Vitamin K2 synthesis, indicating a complex regulatory role in metabolic pathways (13,14). Despite the lack of nitrate respiration in some strains, nitrate reductase genes point to possible conditional expression, necessitating in-depth studies to understand their function under different nitrogen conditions.
- The "Sugar Fermentation Regulator" iModulon, regulated by *CcpA* and *Rex* (15,16), includes genes for alcohol fermentation like *adhP* and *adhE^15,17^* and sugar transporters *rbsK* and *fucP (*18,19), showing *L. reuteri's* carbohydrate versatility. Genes like *LMB90_RS06660*, involved in replication and repair, indicate intricate regulation by sugar levels, pointing to a significant role in energy balance and growth, meriting further investigation.
- The "Intestinal Carbohydrate and Host Interaction" iModulon in *L. reuteri,* with genes like *tadA* for gut adhesion (20) and *xylB* for xylose metabolism (21), suggests a role in host-associated dietary sugar processing. The *rfbD* gene's involvement in rhamnose-rich exopolysaccharide synthesis and immune modulation, especially under calcium ion influence, indicates potential for probiotic benefits (22). Regulated by factors such as *PyrR* and *CcpA*, this iModulon is critical for gut colonization and nutrient metabolism, warranting further research on its role in host interactions (16,23).
- The "Fe-S Cluster Metabolism" iModulon in *L. reuteri*, comprising the *Suf operon* (*sufB, sufD, sufC*) (24) and *feoB (25)*, indicates a role in iron-sulfur cluster assembly and iron assimilation, which is atypical for *L. reuteri's* usual iron-independent lifestyle (26). This finding points to potential metabolic functions or environmental adaptations not previously associated with *L. reuteri*, such as responses to oxidative stress or iron-rich niches, suggesting a complex regulation of metal homeostasis that merits in-depth investigation to uncover its physiological significance.
- The "Oxidative Stress Response" iModulon in *L. reuteri* encompasses genes for oxidative defense (e.g., *LMB90_RS02735*) and energy metabolism (*LMB90_RS04265*), indicating its role in managing oxidative stress and energy needs. It also focuses on protein stability (*LMB90_RS00595*) and is regulated by *CcpA*, reflecting a nuanced response to nutrient status and stressors (16,27). Further studies are needed to clarify its dynamics.
- The "Isoprenoid Biosynthesis and Adaptive Response” iModulon in *L. reuteri* involves genes like *mvaD* and *mvk* for isoprenoid precursors, crucial under stress (28,29,30). It also hints at cell resilience with genes for cell division (*ftsL*) (31) and heat-shock response (*htpX*) (32). Even though isoprenoid biosynthesis systems may be of minor relevance in organisms not known to synthesize isoprenoid, the presence of such systems in *L. reuteri* might reflect uncharacterized metabolic versatility that is not well-studied in *Lactobacillus* (33). However, without direct *in vitro* evidence of isoprenoid biosynthesis in *L. reuteri*, assumptions about this organism’s role in synthesizing natural products are speculative.
- The "Stress Response and Proteostasis” iModulon in *L. reuteri* features genes like *LMB90_RS01580* for coenzyme transport and *LMB90_RS01585* for ion transport and metabolism, suggesting a role in stress response and cellular function. It also includes *grpE* and transcription regulators, hinting at stress adaptation linked to heat shock proteins, with regulation by *HrcA* and *CtsR* (34,35). Further studies are needed to detail this iModulon's role in maintaining protein homeostasis.

**References**

1. Folgering, Joost HA, Paul C. Moe, Gea K. Schuurman-Wolters, Paul Blount, and Bert Poolman. "Lactococcus lactis uses MscL as its principal mechanosensitive channel." *Journal of Biological Chemistry* 280, no. 10 (2005): 8784-8792.
2. Zivkovic, Milica, Marija Miljkovic, Patricia Ruas-Madiedo, Ivana Strahinic, Maja Tolinacki, Natasa Golic, and Milan Kojic. "Exopolysaccharide production and ropy phenotype are determined by two gene clusters in putative probiotic strain Lactobacillus paraplantarum BGCG11." *Applied and Environmental Microbiology* 81, no. 4 (2015): 1387-1396.
3. Senan, Suja, Jashbhai B. Prajapati, and Chaitanya G. Joshi. "Comparative genome-scale analysis of niche-based stress-responsive genes in Lactobacillus helveticus strains." *Genome* 57, no. 4 (2014): 185-192.
4. Hsieh, Pei-Shan, Ching-Wei Chen, Yi-Wei Kuo, and Hsieh-Hsun Ho. "Lactobacillus spp. reduces ethanol‑induced liver oxidative stress and inflammation in a mouse model of alcoholic steatohepatitis." *Experimental and therapeutic medicine* 21, no. 3 (2021): 1-1.
5. Ishida, Minori, Fu Namai, Suguru Shigemori, Shoko Kajikawa, Masami Tsukagoshi, Takashi Sato, Tasuku Ogita, and Takeshi Shimosato. "Ribosome-engineered Lacticaseibacillus rhamnosus strain GG exhibits cell surface glyceraldehyde-3-phosphate dehydrogenase accumulation and enhanced adhesion to human colonic mucin." *Applied and Environmental Microbiology* 86, no. 20 (2020): e01448-20.
6. Pidutti, P., F. Federici, J. Brandi, L. Manna, E. Rizzi, U. Marini, and D. Cecconi. "Purification and characterization of ribosomal proteins L27 and L30 having antimicrobial activity produced by the Lactobacillus salivarius SGL 03." *Journal of applied microbiology* 124, no. 2 (2018): 398-407.
7. Liao, Weilin, Imran Khan, Guoxin Huang, Shengshuang Chen, Liang Liu, Wai Kit Leong, Xiao Ang Li, Jianlin Wu, and W. L. Wendy Hsiao. "Bifidobacterium animalis: the missing link for the cancer-preventive effect of Gynostemma pentaphyllum." *Gut Microbes* 13, no. 1 (2021): 1847629.
8. Chen, Xiabing, Zhiyong Shao, Lijun Wu, Bin He, Wenhai Yang, Jie Chen, Erguang Jin et al. "Involvement of the Actinobacillus pleuropneumoniae ompW Gene in Confrontation of Environmental Pressure." *Frontiers in Veterinary Science* 9 (2022): 846322.
9. Dec, Marta, Renata Urban-Chmiel, Dagmara Stępień-Pyśniak, and Andrzej Wernicki. "Assessment of antibiotic susceptibility in Lactobacillus isolates from chickens." *Gut pathogens* 9, no. 1 (2017): 1-16.
10. Rogosa, M. "Experimental conditions for nitrate reduction by certain strains of the genus Lactobacillus." *Microbiology* 24, no. 3 (1961): 401-408.
11. Xu, Jianlin, and Willy Verstraete. "Evaluation of nitric oxide production by lactobacilli." *Applied microbiology and biotechnology* 56 (2001): 504-507.
12. Maitreya, Anuja, Smita Pal, Asifa Qureshi, Reyed M. Reyed, and Hemant J. Purohit. "Nitric oxide–secreting probiotics as sustainable bio-cleaners for reverse osmosis membrane systems." *Environmental Science and Pollution Research* (2022): 1-19.
13. Bøe, Cathrine Arnason, and Helge Holo. "Engineering Lactococcus lactis for increased vitamin K2 production." *Frontiers in Bioengineering and Biotechnology* 8 (2020): 191.
14. Kang, Min-Ji, Kwang-Rim Baek, Ye-Rim Lee, Geun-Hyung Kim, and Seung-Oh Seo. "Production of vitamin k by wild-type and engineered microorganisms." *Microorganisms* 10, no. 3 (2022): 554.
15. Yang, Xiaopan, Kunling Teng, Rina Su, Lili Li, Tong Zhang, Keqiang Fan, Jie Zhang, and Jin Zhong. "AcrR and Rex control mannitol and sorbitol utilization through their cross-regulation of aldehyde-alcohol dehydrogenase (AdhE) in Lactobacillus plantarum." *Applied and environmental microbiology* 85, no. 4 (2019): e02035-18.
16. Chen, Chen, Linlin Wang, Yanqing Lu, Haiyan Yu, and Huanxiang Tian. "Comparative transcriptional analysis of Lactobacillus plantarum and its ccpA-knockout mutant under galactooligosaccharides and glucose conditions." *Frontiers in Microbiology* 10 (2019): 1584.
17. Peng, Huan, Gang Zhou, Xi-Miao Yang, Guo-Jun Chen, Hai-Bin Chen, Zhen-Lin Liao, Qing-Ping Zhong, Li Wang, Xiang Fang, and Jie Wang. "Transcriptomic Analysis Revealed Antimicrobial Mechanisms of Lactobacillus rhamnosus SCB0119 against Escherichia coli and Staphylococcus aureus." *International Journal of Molecular Sciences* 23, no. 23 (2022): 15159.
18. Cheng, Christopher C., Rebbeca M. Duar, Xiaoxi Lin, Maria Elisa Perez-Munoz, Stephanie Tollenaar, Jee-Hwan Oh, Jan-Peter Van Pijkeren et al. "Ecological importance of cross-feeding of the intermediate metabolite 1, 2-propanediol between bacterial gut symbionts." *Applied and environmental microbiology* 86, no. 11 (2020): e00190-20.
19. Zhao, Yan, Leilei Yu, Fengwei Tian, Jianxin Zhao, Hao Zhang, Wei Chen, and Qixiao Zhai. "An optimized culture medium to isolate Lactobacillus fermentum strains from the human intestinal tract." *Food & Function* 12, no. 15 (2021): 6740-6754.
20. Abdelhamid, Ahmed Ghamry, Samar S. El-Masry, and Noha K. El-Dougdoug. "Probiotic Lactobacillus and Bifidobacterium strains possess safety characteristics, antiviral activities and host adherence factors revealed by genome mining." *Epma Journal* 10 (2019): 337-350.
21. Iliev, Ilia, Tonka Vasileva, Veselin Bivolarski, Albena Momchilova, and Iskra Ivanova. "Metabolic profiling of xylooligosaccharides by Lactobacilli." *Polymers* 12, no. 10 (2020): 2387.
22. Jiang, Yunyun, Min Zhang, Yang Zhang, Justyna Zulewska, and Zhennai Yang. "Calcium (Ca2+)-regulated exopolysaccharide biosynthesis in probiotic Lactobacillus plantarum K25 as analyzed by an omics approach." *Journal of Dairy Science* 104, no. 3 (2021): 2693-2708.
23. Chen, Shaojun, Xinmiao He, Ziliang Qin, Gang Li, Wentao Wang, Zida Nai, Yaguang Tian, Di Liu, and Xinpeng Jiang. "Loss in the antibacterial ability of a pyrr gene regulating pyrimidine biosynthesis after using crispr/cas9-mediated knockout for metabolic engineering in lactobacillus casei." *Microorganisms* 11, no. 10 (2023): 2371.
24. Zhai, Zhengyuan, Yang Yang, Hui Wang, Guohong Wang, Fazheng Ren, Zaigui Li, and Yanling Hao. "Global transcriptomic analysis of Lactobacillus plantarum CAUH2 in response to hydrogen peroxide stress." *Food microbiology* 87 (2020): 103389.
25. Han, Xue, Yuanqiang Tu, Huiying Wu, Lijuan Zhang, and Sainan Zhao. "Gene knockout revealed the role of gene feoA in cell growth and division of Lactobacillus delbrueckii subsp. bulgaricus." *Archives of Microbiology* 203, no. 6 (2021): 3541-3549.
26. Weinberg, Eugene D. "The Lactobacillus anomaly: total iron abstinence." *Perspectives in biology and medicine* 40, no. 4 (1997): 578-583.
27. Zhang, Guofang, Libo Liu, and Chun Li. "Effects of ccpA gene deficiency in Lactobacillus delbrueckii subsp. bulgaricus under aerobic conditions as assessed by proteomic analysis." *Microbial Cell Factories* 19, no. 1 (2020): 1-12.
28. Smeds, Andréas, Tiina Joutsjoki, and Airi Palva. "Identification of a gene cluster for the mevalonate pathway in Lactobacillus helveticus." *DNA Sequence* 12, no. 3 (2001): 187-190.
29. Yang, Min, Fanying Meng, Wen Gu, Lihui Fu, Fan Zhang, Fengjiao Li, Yating Tao et al. "Influence of polysaccharides from Polygonatum kingianum on short-chain fatty acid production and quorum sensing in Lactobacillus faecis." *Frontiers in Microbiology* 12 (2021): 758870.
30. Chamberlain, MaryClaire, Sarah O'Flaherty, Natalia Cobián, and Rodolphe Barrangou. "Metabolomic Analysis of Lactobacillus acidophilus, L. gasseri, L. crispatus, and Lacticaseibacillus rhamnosus Strains in the Presence of Pomegranate Extract." *Frontiers in Microbiology* 13 (2022): 863228.
31. Reverón, Inés, Laura Plaza-Vinuesa, Laura Santamaría, Juan Carlos Oliveros, Blanca de Las Rivas, Rosario Muñoz, and Félix López de Felipe. "Transcriptomic evidence of molecular mechanisms underlying the response of Lactobacillus plantarum WCFS1 to hydroxytyrosol." *Antioxidants* 9, no. 5 (2020): 442.
32. Zhang, Wei, Haifeng Ji, Dongyan Zhang, Hui Liu, Sixin Wang, Jing Wang, and Yamin Wang. "Complete genome sequencing of Lactobacillus plantarum ZLP001, a potential probiotic that enhances intestinal epithelial barrier function and defense against pathogens in pigs." *Frontiers in physiology* 9 (2018): 1689.
33. Fling, Russell R., and Timothy R. Zacharewski. "Aryl hydrocarbon receptor (Ahr) activation by 2, 3, 7, 8-tetrachlorodibenzo-p-dioxin (tcdd) dose-dependently shifts the gut microbiome consistent with the progression of non-alcoholic fatty liver disease." *International Journal of Molecular Sciences* 22, no. 22 (2021): 12431.
34. Van Bokhorst-van de Veen, Hermien, Roger S. Bongers, Michiel Wels, Peter A. Bron, and Michiel Kleerebezem. "Transcriptome signatures of class I and III stress response deregulation in Lactobacillus plantarum reveal pleiotropic adaptation." *Microbial Cell Factories* 12 (2013): 1-15.
35. Rossi, Franca, Teresa Zotta, Lucilla Iacumin, and Anna Reale. "Theoretical insight into the heat shock response (HSR) regulation in Lactobacillus casei and L. rhamnosus." *Journal of theoretical biology* 402 (2016): 21-37.
